# Transcriptomic Perturbations in Placental Gene Expression Following Developmental Exposure to Perfluorooctanoic Acid (PFOA) or Hexafluoropropylene Oxide-Dimer Acid (HFPO–DA or GenX) in CD‑1 Mice Are Consistent with Placental Insufficiency

**DOI:** 10.1021/envhealth.5c00350

**Published:** 2026-03-13

**Authors:** Bevin E. Blake, Vesna A. Chappell, Colette N. Miller, Helen Nguyen, Trina P. Phan, Dhiral P. Phadke, Michele R. Balik-Meisner, Ignacio J. Tripodi, Deepak Mav, Ruchir R. Shah, Suzanne E. Fenton

**Affiliations:** † Public Health and Integrated Toxicology Division, Center for Public Health and Environmental Assessment, Office of Research and Development, U.S. Environmental Protection Agency, Research Triangle Park, North Carolina 27709, United States; ‡ Mechanistic Toxicology Branch, Division of Translational Toxicology, National Institute of Environmental Health Sciences, Research Triangle Park, North Carolina 27709, United States; § Oak Ridge Institute for Science and Education, Center for Public Health and Environmental Assessment, Office of Research and Development, U.S. Environmental Protection Agency, Research Triangle Park, North Carolina 27709, United States; ∥ Sciome LLC, Research Triangle Park, North Carolina 27709, United States; ⊥ Center for Human Health and the Environment, Department of Biological Sciences, North Carolina State University, Raleigh, North Carolina 27695, United States

**Keywords:** PFAS, maternal vascular malperfusion, hypertensive disorders of pregnancy, preeclampsia, placental morphometry

## Abstract

Per- and polyfluoroalkyl substances (PFAS) comprise a group of synthetic chemicals that are ubiquitous global contaminants. Exposure to certain PFAS has been associated with adverse pregnancy outcomes in humans and animal models including preeclampsia and low birth weight. The placenta is critical for a healthy pregnancy, and evidence suggests adverse pregnancy outcomes associated with exposure to certain PFAS may arise from aberrant placental development and/or function. To investigate the effects of two different PFAS on the placenta, samples collected from a prior study of pregnant CD-1 mice exposed to perfluorooctanoic acid (PFOA; 1 or 5 mg/kg) or hexafluoropropylene oxide-dimer acid (HFPO–DA, or GenX; 2 or 10 mg/kg) via oral gavage from embryonic day (E) 1.5 to E 11.5 or E 17.5 were used. Placentas were evaluated for morphometric, transcriptomic, and pathway-level effects. Although relatively few genes were significantly differentially expressed, multiple significantly enriched pathways were identified and were involved in biological processes related to hemostasis (e.g., coagulation and angiogenesis), the innate immune response (e.g., acute phase response signaling), and metabolism, synthesis, and transport of cholesterol, lipids, and bile acids (e.g., FXR/RXR activation and fatty acid metabolism). These pathway-level observations provide a mechanistic basis for observed morphometric changes, fetal:placental weight changes, and previously reported histopathological changes. Overall, these findings confirm proposed Adverse Outcome Pathways consistent with placental insufficiency and fetal nutrient restriction and PFAS-induced maternal vascular malperfusion of the placenta.

## Graphical abstract

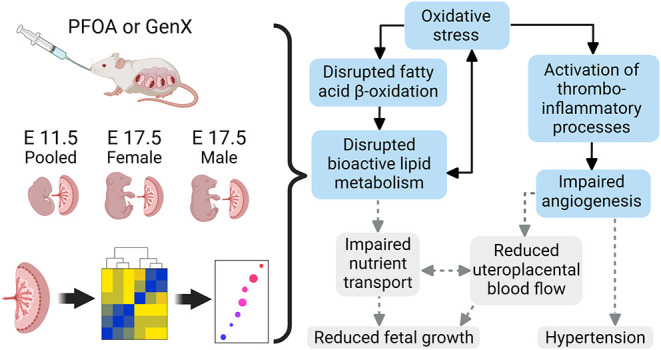

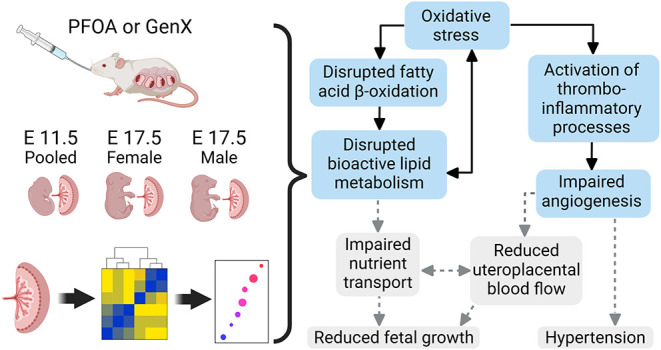

## 1 Introduction

Per- and polyfluoroalkyl substances (PFAS) are a diverse class of chemicals, some of which are used in industry and consumer products to make materials resistant to friction, water, and oil. Exposure to some PFAS subclasses appears ubiquitous in industrialized populations,^1,2^ and previous studies suggest exposure to many PFAS is associated with numerous adverse health effects in humans.^3,4^ Perfluorooctanoic acid (PFOA) is one of the most well-studied PFAS (estimated half-life in humans: 2–4 years, mice: 15–17 days) and exposure to PFOA is associated with adverse pregnancy outcomes including hypertensive disorders of pregnancy (HDP^5–7^ ) and reduced fetal growth or birth weight,^8–11^ among others. Systematic reviews of both the human and animal literature have substantiated the association between maternal exposure to PFOA and reduced fetal growth,^12–14^ which has more recently been estimated to have contributed to approximately 462,000 cases of low birth weight globally per year over the past two decades.^15^ However, exposure to PFAS in the general population occurs as a complex mixture that varies based on many factors including demographics/lifestyle (e.g., high usage of PFAS-containing products), geographical location (e.g., proximity to areas of high PFAS use or production), and temporal variability (e.g., seasonal changes in hydrological processes), among others. PFOA and other legacy PFAS have been implicated in meta-analyses of the impact of PFAS exposure on other adverse pregnancy and birth outcomes, including miscarriage (PFOA;^16^ perfluorodecanoate [PFDA][Bibr ref17]), preterm birth (perfluorooctanesulfonate [PFOS] ^16–18^ ), and low birth weight (PFOS,^16,20^ perfluorononanoic acid [PNFA][Bibr ref19]).

While the underlying mechanism(s) through which exposure to certain PFAS may induce these adverse pregnancy and birth outcomes is not known, it has been posited that the placenta may be an important target and/or mediator.^5,21^ In humans, PFAS can accumulate in the placenta^22–24^ and readily pass through the placenta, with reported transplacental transfer (the ratio of a compound in cord serum to maternal serum) as high as 10.23 for certain PFAS.^25^ Additionally, placental development and function plays an important role in the etiology of adverse pregnancy and birth outcomes, such as hypertensive disorders of pregnancy and fetal growth.^26–28^

While PFOA is no longer produced in the U.S., maternal exposure remains a concern due to its environmental and biological persistence. Replacement compounds, such as hexafluoropropylene oxide-dimer acid (HFPO–DA, also known as GenX), are also of high concern due to widespread human exposure and toxicological effects similar to those of PFOA in animal studies. The Cape Fear River in North Carolina, which serves as a drinking water source for approximately two million residents, was contaminated with GenX between the 1980s and 2017.^29^ Unlike PFOA, the half-life of GenX is much shorter in humans (approximately 81 h) and mice (approximately 16–20 h).^30,31^ Our prior work reported exposure to PFOA or GenX in mice led to increased placental weight, decreased fetal:placental weight ratios, and adverse placental histopathology.^21,32^ However, the underlying mechanism through which PFOA or GenX impacted the placenta is still not understood.

Therefore, this study aimed to investigate the molecular mechanisms and pathways underpinning the relationship between prenatal exposure to PFOA or GenX and placental health using samples previously collected in the study by Blake et al. (2020).^33^ The aims of this study were to (1) identify and compare differentially expressed gene pathways in mouse placenta after gestational exposure to PFOA or GenX by fetal sex and (2) determine whether exposure-modified gene pathways coincide with adverse apical end points observed *in vivo*.

## 2 Methods

### 2.1 Animals

Detailed methods are provided in Appendix A.1 in the Supporting Information file. All tissues were obtained during experiments described in Blake et al. (2020),^21^ which were approved by the NIEHS Animal Care and Use Committee (ASP #2017–0022). Briefly, mice were bred in-house during single overnight cohabitation, and copulatory plug-positive mice were identified as embryonic day (E) 0.5. Pregnant CD-1 mice from the NIEHS colony were singly housed in humidity (45–60%) and temperature (25 °C)-controlled 12 h light cycle rooms in polypropylene cages with *ad libitum* access to NIH-31 diet and reverse osmosis deionized (RODI) water. Pregnant dams (*N* = 10–13 per experimental group) were exposed to 1 or 5 mg/kg perfluorooctanoic acid ammonium salt (PFOA, CAS #3825–26–1, Millipore Sigma), 2 or 10 mg/kg GenX (ammonium 2,3,3,3-tetrafluoro-2- (heptafluoropropoxy) propanoate, CAS# 62037–80–3, SynQuest Laboratories, Alachua, FL, USA), or vehicle (RODI water) via daily oral gavage from embryonic day (E) 1.5 to E 11.5 (when placental vascularization occurs) or E 17.5 (near term when the placenta is fully developed).

### 2.2 Necropsy

Detailed necropsy procedures can be found in Blake et al. (2020)^21^ and in Appendix A.2 in the Supporting Information file. Briefly, on E 11.5 or 17.5, pregnant dams were weighed and humanely euthanized by swift decapitation and trunk blood was collected for internal dosimetry, as reported.^21^ Placentas from viable embryos were collected and immediately snap frozen in liquid nitrogen (*N* = 2–5 placentas/litter). For placentas collected at E 17.5, fetal tissues were genotyped for the sex-determining region Y (SRY) gene to determine fetal sex (TransnetYX, Inc., Cordova, TN, USA). Sex genotyping was not performed for placentas collected at E 11.5 as the reproductive organs are not yet differentiated at this time point.

### 2.3 RNA Isolation and Quantification

RNA isolation and quantification methods are reported in detail in our previous work^33^ and in Appendix A.3 of the Supporting Information file. In summary, a total of *N* = 4–5 placenta per treatment group, time point, and sex (E 17.5 only) were used in the current study. For placentas collected at E 11.5, three placentas per litter were pooled to achieve sufficient tissue mass for the RNA extraction protocol (>0.5 mg, *N* = 4–5 pooled samples per treatment group). For placentas collected at E 17.5, no more than one placenta of each sex was used from a given litter. Briefly, RNeasy kits were used following the manufacturer’s protocols to extract RNA from placenta samples (Cat# 75144, Qiagen, Hilden, Germany). Quality control of the resulting purified RNA was conducted using a NanoDrop 2000/2000c Spectrophotometer (ThermoFisher Scientific, Waltham, MA, USA) and Bioanalyzer High Sensitivity RNA Analysis (Agilent, Santa Clara, CA, USA). RNA expression analysis was conducted using Affymetrix Mouse Clariom D arrays (Affymetrix, Santa Clara, CA, USA).

### 2.4 Gene Expression Data Analyses

A detailed description of the transcriptomic analysis and workflow is described in Blake et al. (2022)^33^ and in Appendix A.4 of the Supporting Information file. Raw data are accessible on NCBI GEO (accession number GSE262609). The probe-level raw intensity signal array was extracted, background adjusted, summarized using robust multiarray average (RMA) technique, and log2 transformed. The normalized expression was averaged across transcripts from the same gene to produce the Entrez gene-level signal. Potential outliers were identified using principal component analysis, hierarchical cluster plots, and correlation plots, resulting in one outlier detected and removed from the analyses. A customized implementation of conventional Gene Set Enrichment Analysis (GSEA)^34^ was employed to simultaneously identify differentially expressed genes (DEGs) and corresponding differentially enriched pathways (DEPs) for each chemical at each dose relative to the corresponding maternal or fetal vehicle control. Student’s *t* test statistics were used to measure gene-level differential activity and perform GSEA tests on all Hallmark pathways from the Molecular Signature Database (MSigDB, version 6.2) for which five or more genes were present in the microarray.^34,35^ In the initial analysis, a gene was required to have an absolute fold change ≥ 2 and a distribution *P*-value ≤ 0.005 to be considered a significant DEG. However, these stringent parameters yielded very few genes, so a secondary analysis was performed using cutoffs of an absolute fold change ≥ 1.5 and a distribution *P*-value ≤ 0.05. A significant DEP required an absolute Normalized Enrichment Score (NES) of ≥ 1.5 and a NES *P*-value of ≤ 0.05.

A comparison analysis was performed using the Ingenuity Pathway Analysis (IPA) Ingenuity Analysis Knowledgebase (Qiagen, Hilden, Germany) to identify similarities and differences in canonical pathways and upstream regulators between the experimental groups and embryonic time points using gene-level results (absolute fold change ≥ 1.5 and *P*-value ≤ 0.05). Fisher’s Exact Test *P*-values identifying significant pathways were then adjusted using the Benjamini-Hochberg multiple testing correction method. Pathways with a −log­(*P*-value) ≥ 1.3 were considered significantly activated.

We then used the DEG set identified using the less stringent criteria (absolute fold change ≥1.5 and *P* ≤ 0.05) to explore the Gene Ontology (GO) Enrichment Analysis (PANTHER version 17.0, GO release 2023–01–01.^36^ The Gene Ontology Consortium, 2020). GO Enrichment Analysis was conducted first using the set of up-regulated DEGs identified across all treatment groups and time points in order to explore the biological processes associated with this suite of genes. GO Enrichment Analysis was conducted a second time using the set of down-regulated DEGs identified across all treatment groups and time points. Both analyses utilized PANTHER Overrepresentation Test using *Mus musculus* as the reference list and Fisher’s Exact test with a False Discovery Rate (FDR) < 0.05.

### 2.5 Verification of Microarray Data

A detailed description of the verification of microarray data is described in Blake et al. (2022)^33^ and in Appendix A.5 of the Supporting Information file. Briefly, RNA from placental samples was used to verify transcriptomic analyses using Real Time PCR (Applied Biosystems, Waltham, MA, USA). Primer sets for genes of interest can be found in Table S1. Relative mRNA levels were determined using the ΔΔCt method and presented as fold change over the respective vehicle control. One-way ANOVA, with Dunnett’s multiple comparisons post hoc test (GraphPad Prism v.7), was performed to determine statistical significance between control and dose groups using ΔCt values (*P*-value ≤ 0.05) and 2-fold change or greater changes are presented as mean fold change for *N* = 4–5, biological replicates, and visualized as a heatmap and p-value table.

### 2.6 Histopathology and Placental Morphometry

Additional detail on the histopathological methods and analyses performed on samples used in the present study are reported thoroughly in Foley et al.^32^ and in Appendix A.6 of the Supporting Information file. Briefly, paraffin-embedded placentas were sectioned and stained with hematoxylin and eosin (H&E) according to standard protocols. Two trained histopathologists evaluated slides and recorded consensus diagnoses.

For the morphometric analyses, only the E 17.5 time point was evaluated. Two trained experimenters blinded to the experimental conditions performed separate morphological assessments for the placental layers and decidual arteries (*N* = 3–6 placentas/group/sex).

### 2.7 Gene Expression-Phenotype Analysis

Additional details for the gene expression-phenotype analysis are reported in Appendix A.7 of the Supporting Information file. To determine whether specific genes or sets of genes were associated with phenotypic changes in the placenta, we performed analyses to evaluate relationships between gene expression and multiple placenta phenotypes. For each E 17.5 placenta with transcriptomic data, we looked at all littermates from the same dam and of the same sex. Continuous phenotypes (fetal weight, placenta weight, ratio of placenta to fetal weight, and placental morphometry) and histopathological severity scores were averaged across littermates.

Phenotypic end points were assessed to directly compare each treatment group to the control group using the many-to-one (treatment groups to control group) Dunn Test with a standard normal distribution, *P*-value ≤ 0.05 was considered significant. Pearson’s product moment correlation test was performed for each gene/phenotype combination to measure the degree of linear dependence between expression and phenotype to assess the relationship between differential gene expression and phenotypeGene-phenotype pairs with correlations ≥ 0.7 and unadjusted *P*-values ≤ 0.05 were considered significant.

## 3 Results

### 3.1 Placental Gene Expression

Transcriptome-wide gene expression analysis was conducted in placental samples collected on embryonic day (E) 11.5 and E 17.5 after maternal exposure to PFOA (1 or 5 mg/kg/day) or GenX (2 or 10 mg/kg/day). Transcriptomic profiles were evaluated using principal component analysis and hierarchical clustering. One outlier in the E 17.5 female GenX 2 mg/kg/day treatment group (sample “21514_1226_Female_Placenta_Low_GenX_2_Rep_2”) was identified and removed from the analysis. After outlier removal, there was a clear separation by time point (E 11.5 vs E 17.5), but there was no obvious separation between treatment groups at E 11.5 and no obvious separation of treatment groups or sex at E 17.5 (Figures S1 and S2).

Gene expression data were first filtered using an absolute fold change ≥2 and *P* ≤ 0.005, which yielded few significant DEGs in the placenta after exposure to PFOA or GenX (Table 1). At this significance threshold, only two DEGs were identified at E 11.5 (*Mir6382*, upregulated, 2 mg/kg GenX) and 3-hydroxy-3-methylglutaryl-Coenzyme A synthase 2 (*Hmgcs2*, upregulated, 10 mg/kg GenX). At E 17.5, a greater number of DEGs were identified in female placenta (15 total DEGs) relative to male placenta (7 total DEGs). In female placentas at E 17.5, significant DEGs identified in PFOA-exposed placentas were all down-regulated and included genes in the eosinophil-associated ribonuclease A family (*Ear12*, *Ear1*) and Ear pseudogenes (*Ear-ps9*, *Ear-ps2*). In female placentas at E 17.5, DEGs identified in GenX-exposed placentas were up-regulated and included CWC22 spliceosome-associated protein, *CWC22*, and seven homologues of this gene (2 mg/kg GenX group only). There were no significant DEGs identified in E 17.5 female placenta exposed to 10 mg/kg GenX. In E 17.5 male placenta, all DEGs were down-regulated and included the Ear genes in the PFOA-exposed groups (1 mg/kg PFOA: *Ear-ps2*, 5 mg/kg PFOA: *Ear12*, *Ear-ps-9*, *Ear1*, *Ear-ps2*) as well as Apolipoprotein A2 (*Apoa2*) in the 5 mg/kg group. In GenX-exposed E 17.5 male placentas, only one significant DEG was identified in the 2 mg/kg group (*Ear-ps2*). There were no significant DEGs identified in E 17.5 male placenta exposed to 10 mg/kg of GenX.

**Table 1:** Number of Significant^a^ Differentially Expressed Genes (DEGs) in Placenta at Embryonic Day (E) 11.5 or 17.5 Following Exposure to PFOA or GenX

**Time point**	**Dose group**	**Up-regulated**	**Down-regulated**	**Total**
E 11.5	1 mg/kg PFOA	0	0	0
E 11.5	5 mg/kg PFOA	0	0	0
E 11.5	2 mg/kg GenX	1	0	1
E 11.5	10 mg/kg GenX	1	0	1
E 17.5 female	1 mg/kg PFOA	0	3	3
E 17.5 female	5 mg/kg PFOA	0	4	4
E 17.5 female	2 mg/kg GenX	8	0	8
E 17.5 female	10 mg/kg GenX	0	0	0
E 17.5 male	1 mg/kg PFOA	0	1	1
E 17.5 male	5 mg/kg PFOA	0	5	5
E 17.5 male	2 mg/kg GenX	0	1	1
E 17.5 male	10 mg/kg GenX	0	0	0

a DEGs were considered significant if absolute fold-change values were ≥2 and *P* ≤ 0.005.

Given the weak clustering of treatment groups and low number of significant DEGs at an absolute fold change ≥2.0 and *P* ≤ 0.005, a less stringent threshold was set in order to explore DEGs at an absolute fold change ≥1.5 and *P* ≤ 0.05. This less stringent analysis yielded 96 total significant DEGs identified in at least one treatment group at either time point (Figure 1 and Table S2). To more broadly explore the biological landscape of these 96 DEGs, Gene Ontology (GO) Enrichment Analysis was conducted. GO Enrichment Analysis of the 65 up-regulated genes yielded 35 hits, of which 33 mapped to biological processes including: Cell killing, Regulation of response to biotic stimulus, Regulation of defense response, Innate immune response, Response to bacterium, and Regulation of response to external stimulus (Table S3). The same analysis conducted on the 31 down-regulated genes yielded 26 hits, of which 25 mapped to biological processes including: response to methanol, response to chromate, negative regulation of serine-type endopeptidase activity, and hydrogen peroxide catabolic process (Table S4).

**Figure 1:**
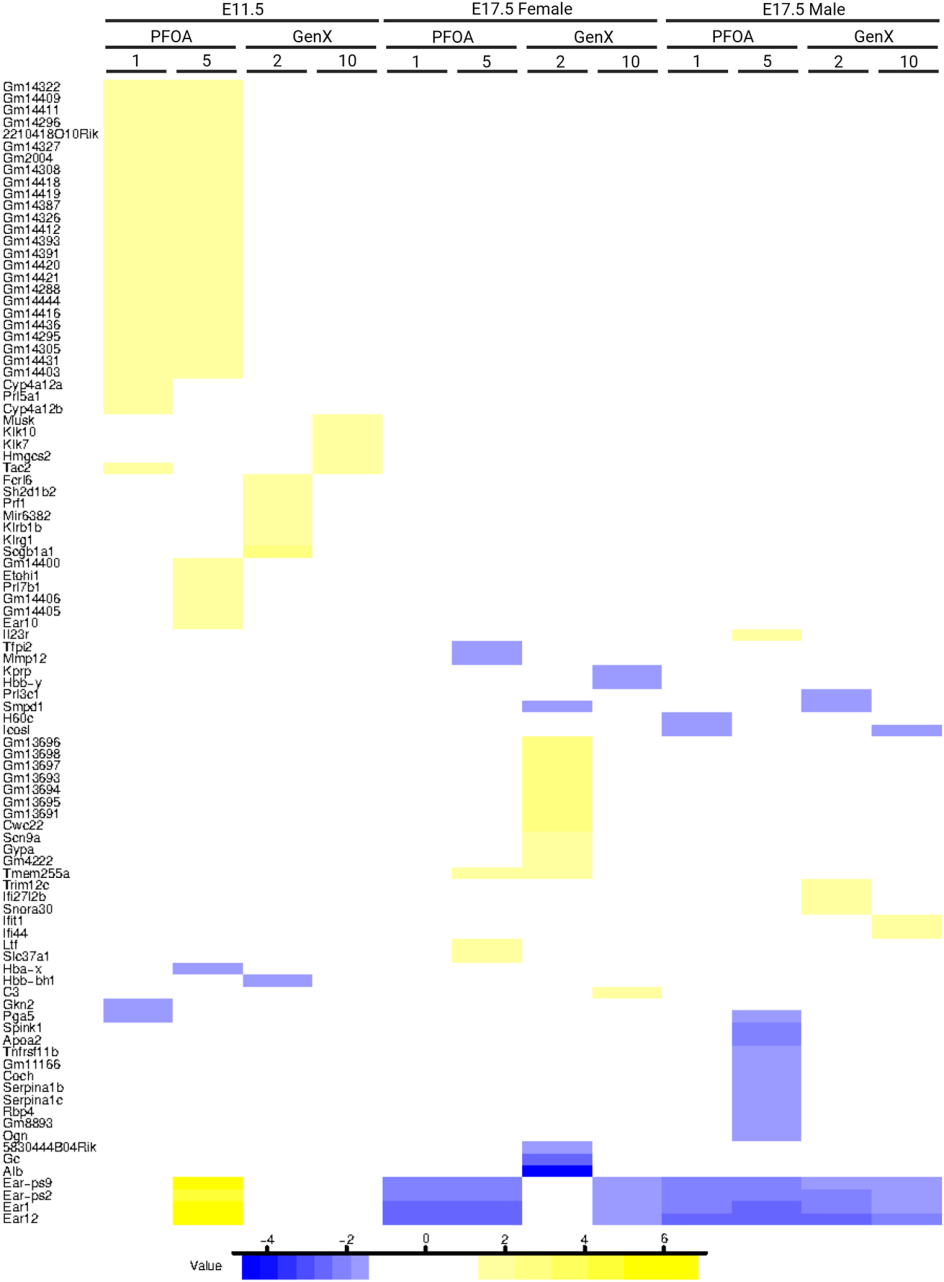
Heatmap indicates significant DEGs identified in placenta after exposure to PFOA (1 or 5 mg/kg) or GenX (2 or 10 mg/kg) at E11.5 and E17.5. Color shading indicates fold-change for the 96 genes with significant expression levels in at least one experimental group, with an absolute fold change of ≥1.5 and *P* ≤ 0.05. White spaces represent comparisons where the gene did not meet the significance threshold.

### 3.2 Verification of Gene Expression by qPCR

Following the transcriptomics analysis, a group of genes underwent further verification by qPCR. The selected genes for verification satisfied one of the two conditions: they displayed either a significant increase (e.g., E 11.5: *Ear1*, *Scgb1a1*; E 17.5: *C3*) or a decrease in expression (e.g., E 11.5: *Gkn2*; E 17.5: *Alb*), or their expression patterns differed between the female (e.g., *GC, Gypa*) and male placenta (e.g., *Rbp4, Serpina1b*) and/or treatment (e.g., GenX: *Smpd1, Prf1*, *Scgb1a1*; PFOA: *Cyp4a12b, Prl5a1*). The verification process confirmed a high level of concordance, with 91% overall agreement between the E 11.5 placenta qPCR gene expression data and the transcriptomics data. A 97% agreement was achieved with respect to expression directionality and 84% agreement of statistically changed genes exhibiting similar expression (qPCR: 1.5-fold change, *P*-value ≤ 0.05; transcriptomics: 2-fold change, *P*-value ≤ 0.005). With respect to the E 17.5 placenta data, a 96% concordance between the qPCR expression data and the transcriptomics data for the established data points falling within the specified thresholds was attained (qPCR: 1.5-fold change, *P*-value ≤ 0.05; transcriptomics: 2-fold change, *P*-value ≤ 0.005). We achieved complete agreement (100%) for directionality and 93% concordance for statistically changed genes exhibiting similar expression (Figure S3 and Table S5).

Expression levels of the prolactin gene family are well characterized spatially and temporally in the mouse placenta, and these genes play important roles during pregnancy; therefore, we used qPCR to further investigate expression levels of prolactin genes which were not captured by our transcriptomics approach. At E 11.5, *Prl5a1* levels were significantly increased in placenta exposed to 1 mg/kg PFOA (Figure 2A). At E 17.5, expression levels of *Prl3a1* in female placenta exposed to 1 or 5 mg/kg PFOA were significantly lower than those in female vehicle controls (*P* ≤ 0.05; Figure 2B). In male placenta at E 17.5, *Prl4a1* expression levels were significantly lower for both 1 mg/kg PFOA and 2 mg/kg GenX, while only *Prl3c1* levels were significantly lower for the 2 mg/kg GenX group (Figure 2C). A significant increase in *Prl6a1* expression levels was observed in male E 17.5 placenta for the 5 mg/kg of PFOA and 10 mg/kg of GenX groups (Figure 2C).

**Figure 2:**
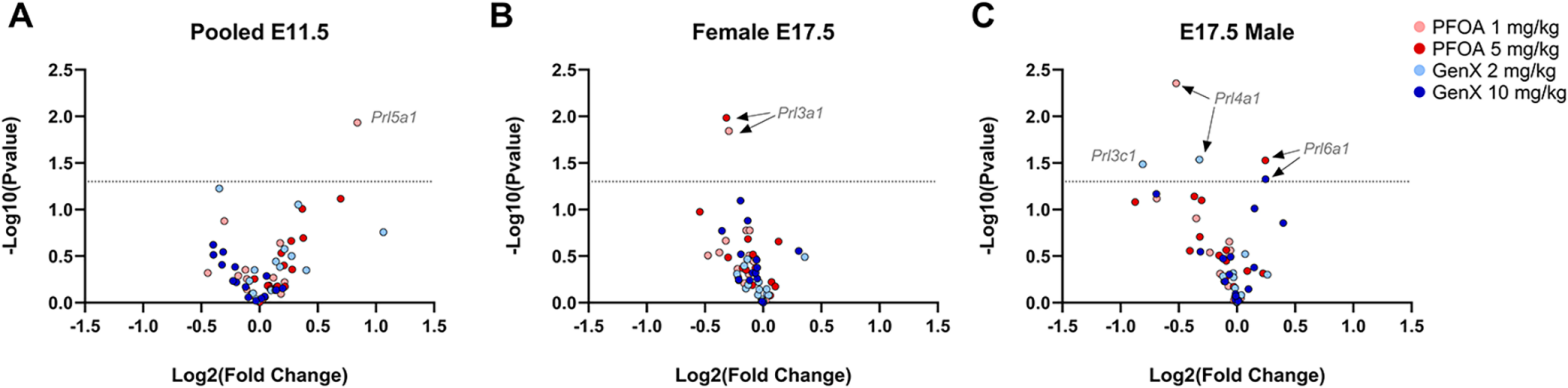
Volcano plots illustrating the perturbations in the distribution of the prolactin gene family in the placenta at (A) E11.5, (B) female placentas at E17.5, and (C) male placentas at E17.5 caused by PFOA and GenX. Transcriptomic data plotted based on −log_10_(*P*-value) as a function of log­(fold change), labeling the statistically significant genes (*P* < 0.05) denoted by gray dotted line.

### 3.3 Pathway Analyses

Significant differentially enriched pathways (DEPs) were detected in all treatment groups across both time points, except E 17.5 male placenta exposed to 1 mg/kg PFOA. In general, placentas exposed to 1 mg/kg PFOA were less sensitive to significant alterations in MSigDB Hallmark Pathway enrichment and placentas at E 11.5 had fewer DEPs than placentas at E 17.5 (Figure 3 and Table S6). Female placentas at E 17.5 exposed to 5 mg/kg PFOA had the greatest number of DEPs, which included: Adipogenesis (up), Fatty acid metabolism (up), Heme metabolism (up), MYC targets V1 (up), MYC targets V2 (up), Oxidative phosphorylation (up), and TGF β signaling (up) (Figure 3 and Table S7). Six DEPs were identified in male placentas at E 17.5 exposed to 5 mg/kg PFOA, none of which overlapped with those identified in female placentas in the same treatment group: Angiogenesis (down), Cholesterol homeostasis (down), Coagulation (down), Epithelial-mesenchymal transition (down), and Estrogen response late (down) (Figure 3 and Table S8). Overall, there was very little overlap within (e.g., high dose vs low dose) and between chemical groups at either time point. Fatty acid metabolism, Heme metabolism, and Peroxisome pathways were all positively enriched in female E 17.5 placentas (*P* ≤ 0.05 for all except Peroxisome in the 5 mg/kg PFOA group, which was borderline significant). For other pathways, the direction of the effect was consistent across groups without reaching statistical significance in every group. For example, epithelial-mesenchymal transition was negatively enriched across all groups at E 17.5 (significant for 5 mg/kg PFOA males and borderline for 10 mg/kg GenX females), and Hypoxia was positively enriched across all groups at E 11.5 (significant for 10 mg/kg GenX and borderline for 2 mg/kg GenX).

**Figure 3:**
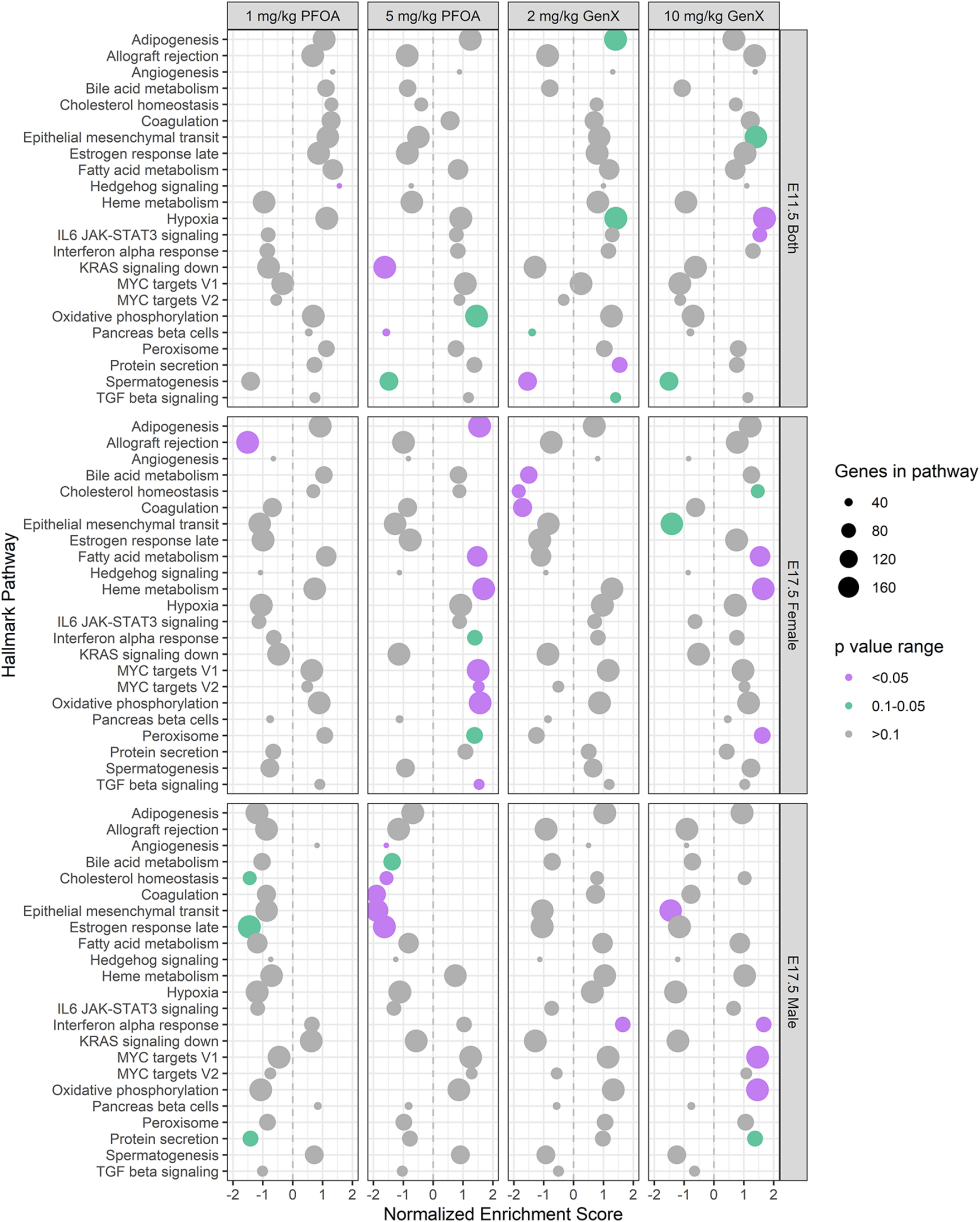
Normalized enrichment scores (NES) for significantly enriched MSigDB Hallmark Pathways in E 11.5 placenta (top panel), E 17.5 female placenta (middle panel), and E 17.5 male placenta (bottom panel). Dot color corresponds to the range of *P*-values associated with the pathway NES (purple: *P* ≤ 0.05, green: 0.05 ≤ *P* ≤ 0.1, and gray: *P* > 0.1). Dot size corresponds to the number of genes in the pathway. Of the 50 MSigDB Hallmark Pathways, only 23 are shown in this figure and represent pathways with a significant NES for at least one time point/treatment group.

Pathway analyses were also performed using IPA and resulted in the identification of numerous significantly enriched pathways with time point and sex-specific patterns. The most common significantly enriched pathways in this analysis included LXR/RXR Activation (2 and 10 mg/kg GenX E 17.5 Female, 5 mg/kg PFOA E 17.5 Male), FXR/RXR Activation (2 and 10 mg/kg GenX E 17.5 Female, 5 mg/kg PFOA E 17.5 Male), Acute Phase Response Signaling (10 mg/kg GenX E 17.5 Female and 5 mg/kg PFOA E 17.5 Male), Erythropoietin Signaling Pathway (5 mg/kg PFOA E 11.5 and 10 mg/kg GenX E 17.5 Female), Iron homeostasis signaling pathway (5 mg/kg PFOA E 11.5 and 10 mg/kg GenX E 17.5 Female), and Sphingomyelin metabolism (2 mg/kg GenX E 17.5 Female and Male, adjusted *P*-value ≤ 0.05; Figure 4A). Upstream regulator analysis in IPA yielded five enriched upstream genes that were significantly enriched in 5 mg/kg PFOA E 11.5 and all E 17.5 dose groups for both sexes except 2 mg/kg GenX E 17.5 Female: Doublesex and mab-3 related transcription factor 1 (DMRT1), C2 Calcium-Dependent Domain Containing 5 (C2CD5), Forkhead Box A3 (FOXA3), Mbt Domain Containing 1 (MBTD1), and Peptidylprolyl Cis/Trans Isomerase, NIMA-Interacting 1 (PIN1; Figure 4B). The greatest number of significantly enriched upstream regulators were identified for the 10 mg/kg GenX E 17.5 Male group (*N* = 38 genes; Figure 4B). The most significantly enriched genes included AIRE (10 mg/kg GenX E 17.5 Male), FZD9 (10 mg/kg GenX E 17.5 Male), and ADAR (10 mg/kg GenX E 17.5 Male; Figure 4B).

**Figure 4:**
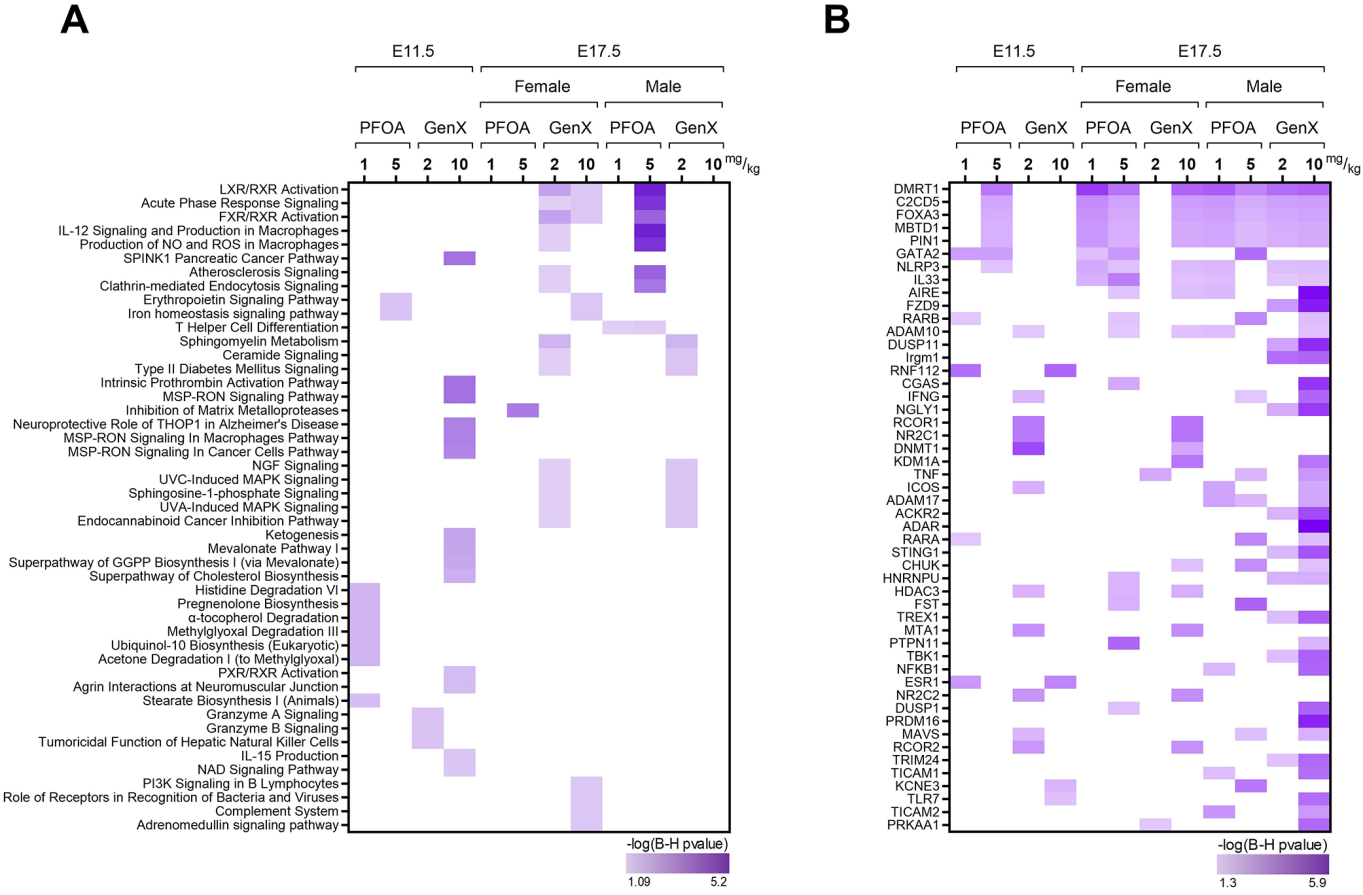
Ingenuity pathway analysis comparison of shared canonical pathways and upstream regulators. (A) Heatmap indicating significantly enriched pathways identified in placenta after exposure to PFOA or GenX at E 11.5 and E 17.5. Color shading indicates −log­(Benjamini-Hochberg adjusted *P-*value), *P ≤ 0.05. White spaces represent comparisons where the pathway did not meet the significance threshold. (B) Heatmap indicating significantly enriched upstream genes identified in placenta after exposure to PFOA or GenX at E 11.5 and E 17.5. Color shading indicates *P* ≤ 0.05 with −log­(Benjamini-Hochberg adjusted *P*-value). White spaces represent comparisons where the pathway did not meet the significance threshold.

### 3.4 Placental Morphometry

Based on preliminary histopathological findings in our earlier report,^21^ a more thorough investigation of adverse histopathological changes in the placenta was conducted by Foley et al.,^32^ who found that treatment-related effects in the placenta were consistent with maternal vascular malperfusion of the placenta. Therefore, morphometric analyses were performed on the placenta collected at E 17.5 to provide deeper insight into the impact of PFAS on the size of the different placental regions (both absolute and relative to total placental area) and spiral arteries (Figure 5A–G). There were no treatment-related effects on the absolute area of the decidua or junctional zone for male and female E 17.5 placenta (Figure 5B, Tables S9 and S10), though it should be noted that Foley et al.^32^ reported increased thickness of the junctional zone. Labyrinth area was significantly increased in the high dose groups for both PFOA and GenX in E 17.5 females and males, while total placenta area was significantly increased in E 17.5 males in the 5 mg/kg PFOA group only (*p* < 0.05, Figure 5B,E,F, Tables S9 and S10). The size of the labyrinth, junctional zone, and decidua relative to the total placenta area was also determined for placenta collected at E 17.5. Relative decidua area was significantly decreased by 32% (females) and 21% (males) in the 10 mg/kg GenX group and 19% (females) and 27% (males) in the 5 mg/kg PFOA group (*P* < 0.05, Figure 5B, Tables S9 and S10). In general, the relative decidua size in treated groups tended to be reduced relative to controls, but this effect did not reach statistical significance in the low-dose groups. There was also a significant 14% increase in relative labyrinth size in E 17.5 female placenta in the 10 mg/kg GenX group (*P* < 0.05, Figure 5B and Table S9) and a significant increase in the ratio of labyrinth to decidua for 10 mg/kg GenX female placenta and 5 mg/kg PFOA male placenta (*P* < 0.05, Figure 5G, Tables S9 and S10).

**Figure 5:**
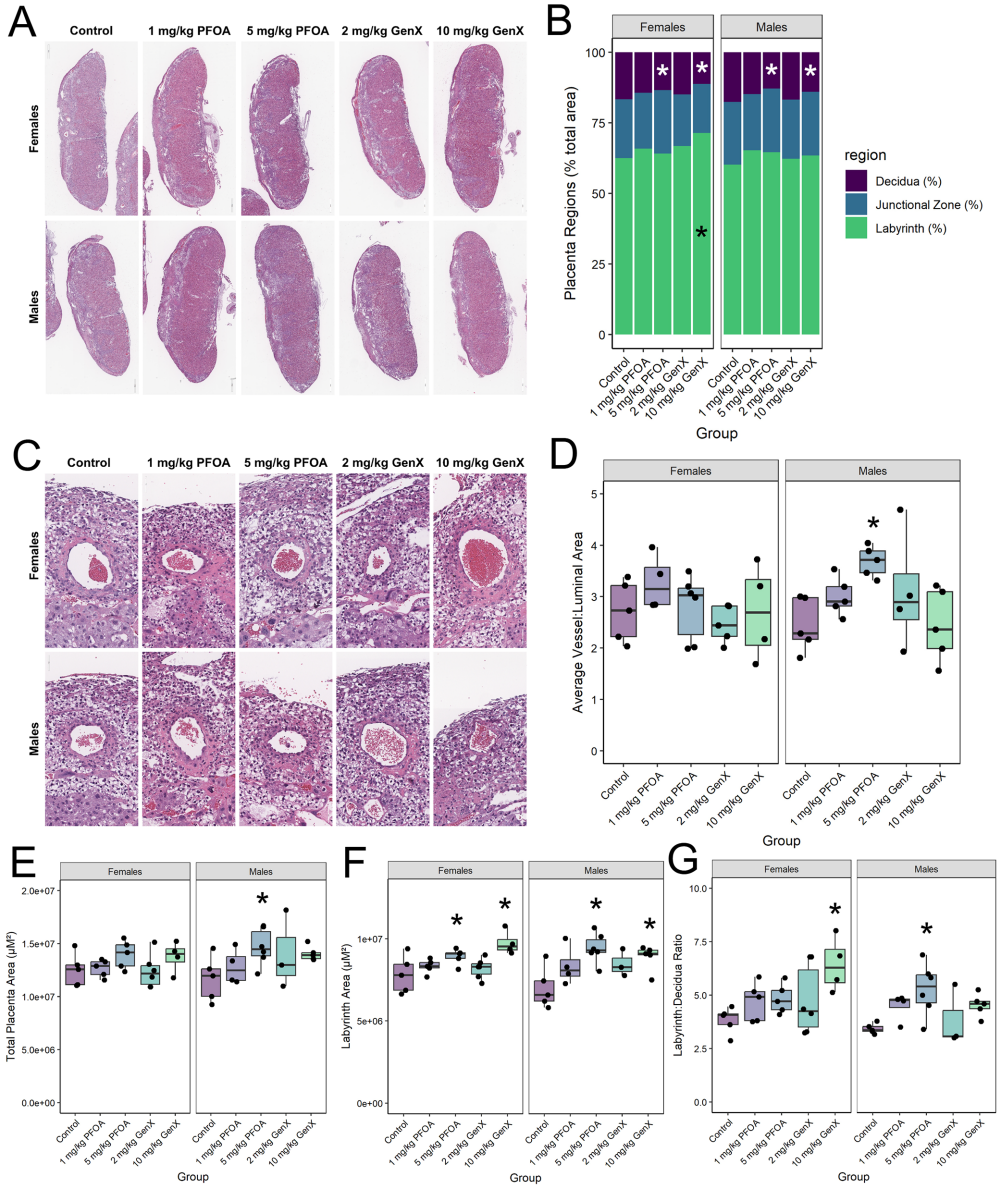
Placental morphometry is altered in male and female placentas following the gestational exposure to PFOA or GenX. (A) Representative cross sections of whole placentas at 5× magnification stained with hematoxylin and eosin (H&E). (B) Relative area of the placental decidua, junctional zone, and labyrinth shown as a percentage of total placenta area. (C) Representative images of the maternal spiral arteries at 40× magnification stained with H&E. (D) Average vessel area to average luminal area of the spiral arteries. (E) Total placenta area, (F) labyrinth area, (G) ratio of the labyrinth area to the decidua area. *P < 0.05 ANOVA with post hoc correction using Dunn Test. Image created using BioRender.

The luminal area and total vessel area, inclusive of the arterial wall, were determined, and the ratio between the two was calculated as an estimate of impaired arterial remodeling. There was a significant increase in the ratio of average vessel to luminal area for 5 mg/kg PFOA male placenta (*P* < 0.05, Figure 5C,D and Table S10). There were no significant treatment-related effects for the average vessel area, average luminal area, and average luminal length (Tables S9 and S10).

### 3.5 Gene Expression-Phenotype Analysis

To determine whether specific genes or sets of genes were associated with phenotypic changes in the placenta, we performed correlation analyses (for fetal weight, placental weight, fetal:placental weight ratios, morphometry, and histopathology).

Overall, for E 17.5 female placenta, there were 451 genes significantly correlated with at least one phenotypic end point, while for E 17.5 male placenta, there were 603 significantly correlated genes (Table S11). For E 17.5 female placenta, end points with the greatest number of significantly correlated genes included coagulative necrosis of spongiotrophoblasts in the junctional zone (*N* = 109), fetal:placental weight ratio (*N* = 45), average vessel luminal area (*N* = 37), angiectasis of the junctional zone (*N* = 31), labyrinth congestion (*N* = 24), and glycogen cell lacunae with minimal acellular contents (*N* = 24; Table S11). For E 17.5 male placenta, end points with the greatest number of significantly correlated genes included decidua to junctional zone ratio (*N* = 104), relative decidua area (*N* = 61), coagulative necrosis of spongiotrophoblasts in the junctional zone (*N* = 56), glycogen cell lacunae with acellular basophilic contents in the junctional zone (*N* = 32), and angiectasis in the junctional zone (*N* = 32, Table S11). Of the 104 genes significantly correlated with decidua to junctional zone ratio in male placenta, 3 of these genes overlapped with the group of DEGs identified using less stringent criteria (fold ≥1.5 and *P* ≤ 0.05) and included *Apoa2*, *Serpina1b*, and *Gm8893* (Figure 5A–C; Table S11). Of the 8 genes significantly correlated with decidua necrosis in E 17.5 males, only *Fam189a2* was identified as a DEG (Figure 6D; Table S11). For the E 17.5 female placenta, three of the 15 genes significantly correlated with maternal sinus dilation were identified as DEGS: *2310036O22Rik*, *Gm13316*, and *Met* (Figure 6E–G; Table S11).

**Figure 6:**
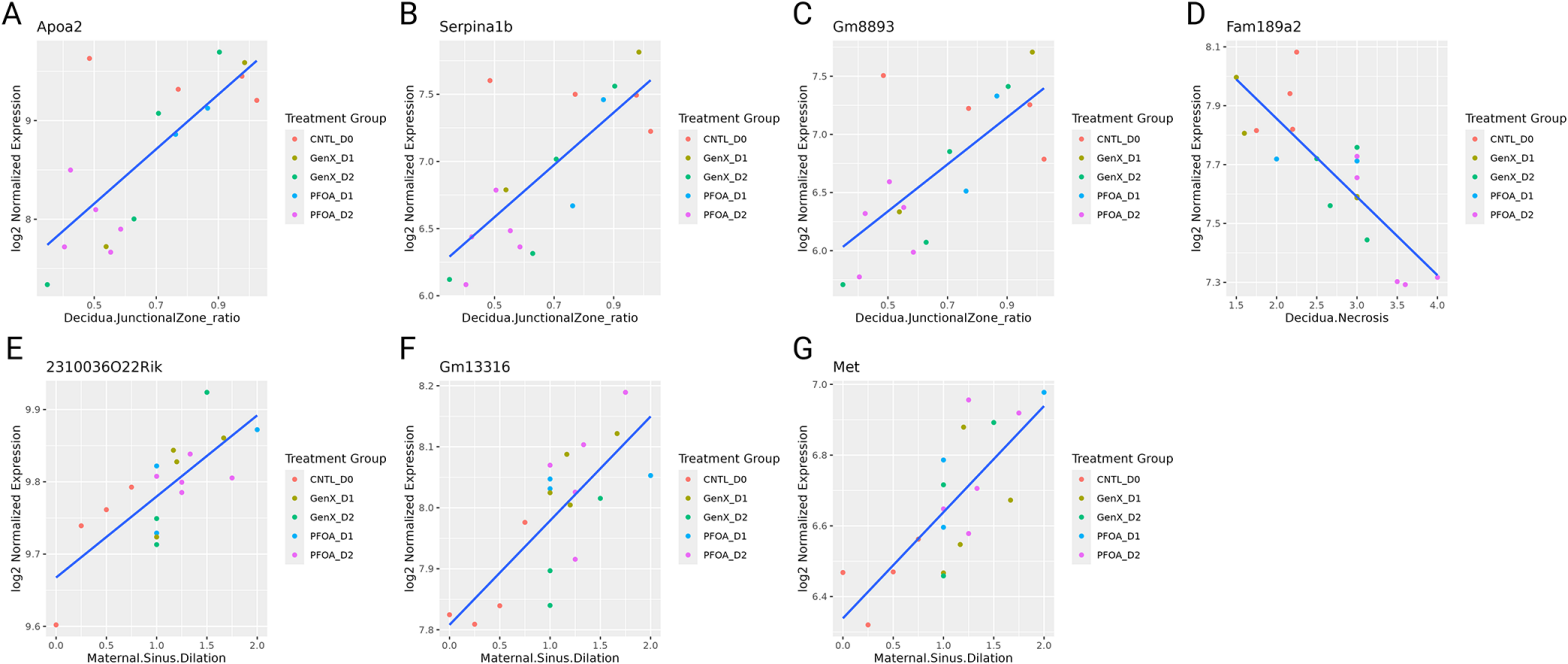
DEGs significantly correlated with placenta end points in E17.5 male (A–D) and female (E–G) placenta following gestational exposure to PFOA or GenX. Gene expression of (A) *Apoa2*, (B) *Serpina1b*, and (C) *Gm8893* was positively correlated with the ratio of decidua to junctional zone area, with higher expression levels and ratios in E17.5 male controls. Gene expression of (D) *Fam189a2* was negatively correlated with decidua necrosis, with higher expression levels and lower decidua necrosis in E17.5 male controls. Gene expression of (E) *2310036O22Rik*, (F) *Gm13316*, and (G) *Met* was positively correlated with maternal sinus dilation, with lower expression levels and lower maternal sinus dilation in E17.5 female controls.

### 3.6 Quantitative Adverse Outcome Pathway (qAOP) Analysis

We assessed the three recently published putative AOPs for neonatal mortality (AOP 1), low birth weight (LBW)/fetal growth restriction (FGR) (AOP 2), and neonatal mortality (AOP 3) (described in Rogers et al. 2023 ^37^) for significant enrichment by the transcriptome-wide placental gene expression changes reported here (Figure 7). For AOP 1, there was significant enrichment of three out of four molecular initiating events: PPARalpha activation (E 11.5 1 mg/kg PFOA; E 11.5 10 mg/kg GenX; E 17.5 Female 1 mg/kg PFOA), PPARgamma activation (E 11.5 2 mg/kg GenX; E 11.5 10 mg/kg GenX; E 17.5 Female 2 mg/kg GenX; E 17.5 Female 10 mg/kg GenX; E 17.5 Female 1 mg/kg PFOA; E 17.5 Female 5 mg/kg PFOA), and CAR/PXR activation (E 17.5 Female 1 mg/kg PFOA; E 17.5 5 mg/kg PFOA; Figure 7). Cell and organ level key events with significant enrichment in AOP 1 included decreased trophoblast invasion and angiogenesis (E 11.5 1 mg/kg PFOA, 5 mg/kg PFOA, 2 mg/kg GenX, 10 mg/kg GenX; E 17.5 Female 2 mg/kg GenX), and perinatal hypoglycemia (E 17.5 Female 2 and 10 mg/kg GenX; E 17.5 Male 2 mg/kg GenX; Figure 7). AOP 2 shared several MIEs with AOP 1 (CAR/PXR activation, PPARalpha and γ activation) and was significantly enriched in a separate MIE unique to AOP 2: transthyrethrin binding (E 11.5 1 mg/kg PFOA; E 11.5 10 mg/kg GenX; E 17.5 Female 1 mg/kg PFOA). AOP 3 was significantly enriched at both MIEs: Impaired pulmonary surfactant function (E 17.5 F 5 mg/kg PFOA) and PPARgamma downregulation (E 11.5 5 mg/kg PFOA; E 11.5 2 mg/kg GenX; E 17.5 Female 1 mg/kg PFOA, 5 mg/kg PFOA, 2 mg/kg GenX, 10 mg/kg GenX; Figure 7).

**Figure 7:**
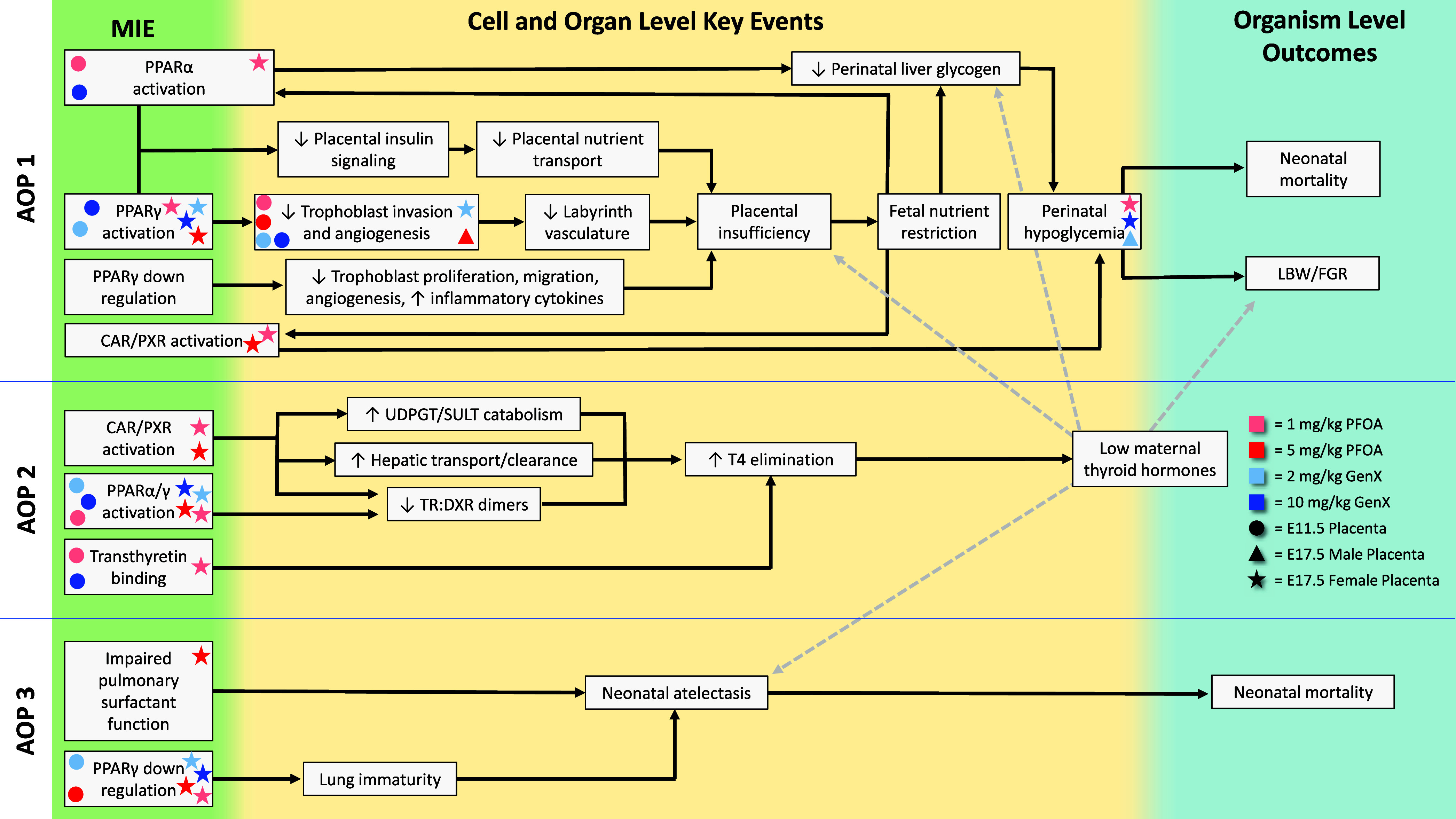
Mapping of transcriptomic data to the AOP network proposed by Rogers et al. (2023). Molecular initiating events and Key Events are marked with symbols to indicate significant enrichment of the AOP component by placental transcriptomic results. Pink symbols = 1 mg/kg PFOA, red symbols = 5 mg/kg PFOA, light blue symbols = 2 mg/kg GenX, and dark blue symbols = 10 mg/kg GenX. Circles = pooled E 11.5 placenta, triangles = E 17.5 male placenta, and stars = E 17.5 female placenta. Figure adapted with permission from Rogers et al. (2023) 2023 The Authors. *Birth Defects Research* is published by Wiley Periodicals LLC.

## 4 Discussion

Placental transcriptomic data were evaluated in samples collected during the original study by Blake et al. (2020)^21^ following gestational exposure to PFOA (1 or 5 mg/kg/day) or GenX (2 or 10 mg/kg/day) from E 1.5 through E 11.5 and E 17.5 in order to determine molecular pathways underlying previously observed phenotypic changes, including altered fetal-placental weight ratios and histopathological changes consistent with maternal vascular malperfusion in the placenta.^21,32^ Although a relatively low number of individual significant DEGs were identified, placental morphometry, pathway analyses, and gene expression-phenotype correlation analyses revealed developmental time point and sex-specific placental responses to gestational exposure to PFOA or GenX. Furthermore, we mapped the transcriptomic data to a putative adverse outcome pathway (AOP) network for neonatal mortality and lower birth weight proposed by Rogers et al. (2023)^37^ and found significant enrichment of molecular initiating events (MIE) for all three AOPs in the network. Taken together, the data presented herein provide insight into the underlying biological pathways disrupted in the placenta following gestational exposure to two different PFAS.

Exposure to certain PFAS in humans is associated with increased risk for numerous adverse pregnancy outcomes including hypertensive disorders of pregnancy (HDP) such as preeclampsia. Preeclampsia is a complex pregnancy condition thought to represent a heterogeneous syndrome resulting from multiple pathogenic pathways.^38^ One hypothesized pathway is excess hypercoagulation leading to microthrombi in placental circulation and decreased placental perfusion, resulting in maternal hypertension and proteinuria.^39^ In healthy pregnancies, various hemostatic and inflammatory mediators interact to generate a state of hypercoagulability.^40,41^ A study in humans comparing the proteomic profile of maternal serum in early onset severe preeclamptic to uncomplicated pregnancies demonstrated that the complement and coagulation cascades were the main differentially enriched pathways.^42^ Here we report dysregulation of numerous pathways related to hemostasis (e.g., coagulation Hallmark Pathway and atherosclerosis, erythropoietin signaling, intrinsic prothrombin activation, and complement system Canonical Pathways) in placenta after gestational exposure to PFOA or GenX. These pathway changes were coherent with the observed maternal vascular malperfusion phenotype reported in Foley et al.^32^ It is possible that gestational exposure to certain PFAS increases risk for hypertensive disorders of pregnancy such as preeclampsia through disruption of hemostatic processes.

Hypercoagulability and low-grade inflammation are among the many physiological adaptations that occur during normal pregnancy and disruptions in hemostasis and inflammatory responses (also called thrombo-inflammatory processes) have been implicated in the etiology of adverse pregnancy outcomes including preeclampsia and intrauterine growth restriction.^40,43^ Specifically, placental trophoblast activation of platelets, which in turn activates pro-inflammatory factors, may play a critical role in mediating thrombo-inflammatory processes implicated in HDP.^40^ Our data support the hypothesis that complex thrombo-inflammatory interactions involving hemostasis and the innate immune system may be perturbed by gestational exposure to certain PFAS. In addition to providing evidence for disrupted hemostatic pathways, we also identified disrupted pathways in the innate immune response (e.g., IL-12 signaling and production in macrophages, acute phase response signaling) in the mouse placenta following exposure to PFOA or GenX, which may indicate immune maladaptation in response to the exposure. Furthermore, IL-33 and NLRP3 were identified as significant upstream regulators in all exposure groups at E 17.5 except for the 2 mg/kg GenX female group. Prior work has suggested that activation of the NLRP3 inflammasome inhibits IL-33 signaling in preeclampsia, resulting from disruption of abnormal vascular remodeling during early pregnancy.^44,45^ In a human pregnancy cohort, total serum PFAS levels were found to be significantly inversely associated with numerous hematological biomarkers with specific roles in the immune system, including platelets.^46^ Given that several well-studied PFAS are considered immunotoxicants,^47,48^ the link between maternal levels of these are other PFAS, thrombo-inflammatory processes, and birth outcomes is a critical data gap warranting further exploration.

The placental prolactin gene family (*Prl*) plays a critical role in the establishment and maintenance of healthy pregnancy, including vascular remodeling, hematopoiesis, and immune regulation of uterine natural killer cells.^49^ Additionally, placental prolactin plays an important role in the epithelial-mesenchymal transition (EMT) of extravillous trophoblasts, which facilitates the ability of trophoblasts to invade the decidua.^50,51^ In the present study, the EMT pathway was found to have decreased enrichment in E 17.5 male placenta in the 5 mg/kg PFOA group. Further, we identified alterations in the expression level of several members of the prolactin gene family, including decreased expression of *Prl3a1* (E 17.5 female 1 and 5 mg/kg PFOA), *Prl3c1* (E 17.5 male 2 mg/kg GenX) and *Prl4a1* (E 17.5 male 1 mg/kg PFOA and 2 mg/kg GenX) and increased expression of *Prl5a1* (E 11.5 1 mg/kg GenX) and *Prl6a1* (E 17.5 male 5 mg/kg PFOA and 10 mg/kg GenX). *Prl4a1* is thought to play an important role in regulating pregnancy adaptations in response to hypoxia.^52,53^ Prior work in humans have shown reduced expression of *PRL3A1* in placentas of humans born small for gestational age,^54^ while *Prl3a1* levels were reduced in term placenta in nutrient-restricted mice.^55^ *Prl5a1* levels were increased at E 11.5 (1 mg/kg PFOA), which may reflect a compensatory change as *Prl5a1* is a specific marker of intrauterine invasive trophoblast cells.^56^

We observed significant correlations between gene expression and placental phenotypes in male and female E 17.5 placenta including several genes that were also identified as significant DEGs. In E 17.5 males, serine peptidase inhibitor, clade A, member 1B (*Serpina1b*) and apolipoprotein A2 (*Apoa2*) expression levels were positively correlated with the ratio of decidua to junctional zone, with exposed males having lower levels of expression and lower ratios compared with control males. In a study of human pregnancies, protein and mRNA levels of SERPINA1 were decreased on the maternal side of the placenta in spontaneous preterm births^57^ while other studies have found that increased and aberrant urinary SERPINA1 peptides were associated with preeclampsia.^58,59^ The link between placenta *Serpina1* expression and urinary SERPINA1 peptides is not entirely clear, but it has been hypothesized that the serine protease inhibitor (SERPIN) gene family is induced by hypoxia and expression levels are modified in preeclamptic placentas.^60^ Studies of nutrient restriction in rats^61^ and of intrahepatic cholestasis of pregnancy in women^62^ have shown up-regulation of the *Apoa2* gene and protein, respectively. A study of women with gestational diabetes mellitus, however, found plasma levels of APOA2 were significantly lower than controls.^63^ While our study showed exposed animals had decreased expression of *Apoa2*, coinciding with decreased decidua to junctional zone ratios, it is possible that this gene expression pattern may reflect a compensatory mechanism occurring in late gestation. Regardless, the expression and activity of apolipoproteins play a critical role in placental cholesterol transport and is downstream of LXR/RXR and FXR/RXR activation,^61^ which we found to be significantly enriched by the gene set in the GenX-exposed E 17.5 female placenta and 5 mg/kg PFOA E 17.5 male placenta. The biological significance of the correlation between expression levels of genes like *Apoa2* and *Serpina1b* and the ratio of decidua to junctional zone is not yet clear, however in a reduced uterine perfusion pressure mouse model of preeclampsia, the junctional zone was found to be significantly larger than in control animals.^64^

Aberrant hypoxia may be an important mechanism through which certain PFAS induce placental alterations. We observed consistent positive normalized enrichment scores for the Hypoxia Hallmark pathway in placenta at E 11.5, though only the 10 mg/kg GenX group reached statistical significance. Under normal circumstances, hypoxia serves as an essential signaling mechanism that directs trophoblast cell differentiation, placentation, and placental development (reviewed by Soares et al. 2018 ^65^). Thus, even small disruptions of hypoxia levels in the developing placenta may be biologically significant to the developing fetus. As discussed, prolactin family genes and SERPIN family genes regulate expression levels in response to placental hypoxia and were significantly altered in this study. Expression levels of MET proto-oncogene, receptor tyrosine kinase (*Met*) were significantly correlated with the severity of maternal sinus dilation in female E17.5 placenta, with controls exhibiting low expression levels and low incidence of sinus dilation. During placental development, Met serves as a receptor for hepatocyte growth factor (HGF), a critical regulator of placental development involved in trophoblast migration, invasion, and vascular remodeling. A study of *ex vivo* human placental cells provided evidence for the dysregulation of HGF/Met signaling under hypoxic conditions^49^ while other studies have demonstrated a link between dysregulation of the Met/ERK pathway (downstream of HGF/Met) and preeclampsia.^66–68^

Fatty acid uptake, metabolism, and oxidation in the placenta are critical to fuel its energetic demands at all stages of development, and disruptions in mitochondrial fatty acid oxidation (FAO) are associated with preeclampsia and HELLP syndrome (hemolysis, elevated liver enzymes, and low platelets).^69^ Fetal growth and development can be altered by decreased placental fatty acid β-oxidation, leading to intracellular lipid accumulation, which perturbs the transport of fatty acids to the developing fetus.^70^ In mice exposed to a high-fat diet during pregnancy, placental FAO gene expression was disrupted, placental weights were increased, and fetal-placental weight ratios were decreased.^71^ In our prior work, we reported increased placental weights and decreased fetal-placental weight ratios following gestational exposure to PFOA or GenX,^21^ and in the present work, we show alterations in Hallmark pathways related to fatty acid metabolism, cholesterol homeostasis, oxidative phosphorylation, and peroxisomes. Similarly, gestational exposure to perfluorohexane sulfonate (PFHxS) in mice resulted in disrupted placental lipid homeostasis as well as histopathological changes in the placental labyrinth.^72^ It is likely that PFAS disrupts lipid and fatty acid metabolism across the maternal liver-placenta-fetal liver axis, reducing the capacity of the placenta to appropriately deliver nutrients to the developing fetus. Indeed, a metabolomics study in adolescents identified dysregulated lipid metabolism, increased lipolysis, and β-oxidation as underlying mechanisms through which exposure to certain PFAS elicits adverse cardiometabolic effects.^73^ Similarly, mouse offspring gestationally exposed to GenX exhibited increased fat mass, insulin sensitivity, and hepatocellular microvesicular fatty change in the liver.^74^ In rats, gestational exposure to GenX resulted in liver gene expression changes, reduced liver glycogen, and other outcomes consistent with disrupted lipid and fatty acid metabolism in the offspring and dams.^75–77^ Similar findings have been reported in rats following gestational exposure to PFOS, PFOA, PFMOAA, POFO5DoA, and PFO4DA.^78–81^ PFAS-induced lipid dysfunction has also been mechanistically corroborated by *in vitro* studies of placental trophoblasts, summarized in the review by Szilagyi et al. (2020).^82^ Disrupted lipid homeostasis during pregnancy may have long-lasting effects on the cardiometabolic health of the offspring. Our prior work demonstrated adverse metabolic outcomes in the offspring with males being particularly affected.^74^ The present work provides further insight into the mechanisms through which certain PFAS may induce developmental maladaptive programming of cardiometabolic health.

The placenta itself is a critical modulator of bile acid transport between maternal and fetal circulation, and it has been hypothesized that the functional effects of bile acids may activate sphingosine-1-phosphate (S1P) receptors to improve nutrient delivery to the fetus, thereby supporting placental and fetal development.^83^ Sphingolipids (such as S1P) are bioactive lipids with important roles in vascular homeostasis, and disruptions in sphingolipid synthesis and metabolism have been implicated in preeclampsia pathogenesis in mice^84^ and humans.^85^ Here we show that gestational exposure to PFOA or GenX disrupted placental sphingomyelin metabolism, S1P signaling, ceramide signaling, FXR/RXR activation, and bile acid metabolism. Our findings parallel those reported in mice following gestational exposure to PFHxS; in addition to reporting decreased placental labyrinth zone area and thickness, decreased placental sinusoid area, and altered fetal:placental weight ratios, PFHxS exposure also disrupted placental lipidomics, including sphingomyelins (among other lipid classes) and altered metabolic pathways that included phospholipid biosynthesis, steroid biosynthesis, and glycerolipid metabolism.^72^ In a human cohort study, cord blood levels of PFAS were associated with dysregulation of multiple lipid classes as well as bile acids.^86^ Based on their findings, Sinisalu et al. (2021) hypothesized that the mechanism through which certain PFAS disrupt lipid metabolism may also occur through bile acid activation of the farnesoid X receptor (FXR).^86^ FXR is considered a bile acid sensor and activation of FXR has been shown to protect the placenta from oxidative stress caused by maternal cholestasis in mice.^87^ Studies of PFOA, PFOS, PFO4DA, and PFO5DoA have shown increased levels of fetal bile acids following gestational exposure in rats.^79–81^ Taken together, these findings suggest certain PFAS may alter the entero-hepatoplacental bile acid axis during pregnancy.

The placenta is a sexually dimorphic organ, and fetal sex is associated with variable risk levels for adverse pregnancy outcomes. In their systematic review and meta-analysis, Broere-Brown et al. (2020) reported male fetal sex was associated with an increased risk for preeclampsia and gestational hypertension and hypothesized this could be due to the higher cardiovascular and metabolic load experienced during pregnancy with a male fetus.^88^ Another explanation for the higher risk of pregnancy complications associated with male fetal sex is sex-specific differences in the immune response during pregnancy. Pregnancies with male fetuses have higher levels of inflammatory immune responses (reviewed in Baines and West 2023 ^89^). Here, we observed sex differences in placenta at E 17.5 with males in the 5 mg/kg PFOA group showing the greatest number of significantly altered pathways and E17.5 male placenta having a greater number of genes significantly correlated with histopathological or morphological changes compared with females. However, it should be noted that the variability in response between chemicals, doses, and developmental time points was similar to the level of variability in response between sexes. Additionally, robust sex differences in placental morphometry and histopathology (reported in Foley et al.^32^) were not apparent. While sex differences should not be discounted in studies of the placenta, sex differences were not robust in the present work. Further studies are needed to improve our understanding of the sex differences in placental susceptibility to PFAS exposure.

Several of the significantly enriched upstream regulators identified in our analysis point to disruptions in early placental development as potential causes of the observed tissue- and organ-level sequalae. For example, Malignant Brain Tumor Domain-containing Protein 1 (MBTD1), plays a critical role in endometrial stromal cell decidualization^90^ while peptidyl-prolyl cis/trans isomerase (PIN1) knockdown in mice impairs implantation and decidualization, and PIN1 inactivation has been reported in preeclamptic human placentas.^91,92^ *In vitro*, Forkhead Box O3A (FOXA3) protein has been shown to play a role in regulating trophoblast invasion and migration during placental development,^93^ while *in vivo* studies have shown GATA2 is crucial for syncytiotrophoblast development^94^ and circulating GATA2 mRNA levels are decreased in preeclamptic pregnancies.^95^ These early disruptions in placental development are consistent with the finding of a significant negative normalized enrichment score for EMT observed in the 5 mg/kg of PFOA and 10 mg/kg GenX E17.5 male groups. During EMT in early placental development, extravillous trophoblasts transition from an epithelial phenotype into a migratory/invasive mesenchymal phenotype, which allows them to remodel the maternal decidua and vessels.^96^ Thus, dysregulated EMT has been implicated in the pathogenesis of pre-eclampsia and fetal growth restriction. While this evidence requires further interrogation, it may provide a useful starting point for future studies aimed at delving deeper into the mechanistic origins of placental perturbations induced by certain PFAS.

Overall, these data represent an explorative study of placental transcriptome-wide gene expression changes after gestational exposure to PFOA or GenX. Although validation studies are needed for some of the specific findings, the results suggest PFOA and GenX disrupt placental pathways involved in cholesterol and lipid nutrient transport, the innate immune response (specifically inflammation), and hemostasis (specifically blood coagulation systems), ultimately resulting in a phenotype consistent with maternal vascular malperfusion of the placenta. These pathways have translational relevance to human pregnancy conditions previously associated with maternal exposure to certain PFAS, including preeclampsia and other hypertensive disorders of pregnancy, and suggest a putative toxicological mechanism through which certain PFAS exert adverse effects on the maternal-placental-fetal unit.

There are several limitations to the work presented here. As noted in our prior work, the sample sizes are relatively small (*N* = 4–5 per treatment group, time point, and sex). Despite the small sample size, we were still able to detect significant effects, particularly using the pathway, gene expression-phenotype, and other correlation analyses. A larger sample size would increase confidence in these findings, but our results are consistent with our prior work as well as studies conducted by other groups (e.g., Yao et al. 2023 ^72^). As with our prior work, an important strength of this study is that the transcriptomic and pathway analyses are supported by robust phenotypic and histopathological data collected from the same cohort of animals. A potential limitation of the current work is the relatively modest extent to which individual genes were differentially expressed, particularly in comparison to the level of effect observed in our prior transcriptomics study using maternal and fetal liver.^33^ It is likely the placenta has more compensatory capability than the liver and/or is more resistant to gene expression changes than the liver or that more profound PFAS-induced alterations in the placenta occur downstream from gene expression changes. Future work should consider incorporating multiomics (e.g., metabolomics, lipidomics) into experiments designed to evaluate placental toxicity of PFAS or utilize spatial transcriptomics to identify the areas (cells) which are the primary developmental targets.

## 5 Conclusions

In this study of gene expression, pathway, and morphometric changes in the mouse placenta following gestational exposure to PFOA or GenX, we found that pathway-level effects were coherent with alterations in placental morphometry (e.g., altered spiral arteries, decreased decidua, increased labyrinth to decidua ratios) and histopathological analyses reported by Foley et al.^32^ (e.g., decidual necrosis, fibrinoid necrosis of the spiral artery, labyrinth congestion). Pathway analyses provide an improved understanding of the mechanisms underlying these tissue-level effects and included biological processes related to hemostasis, the innate immune response, fatty acid uptake, metabolism, and oxidation, cholesterol homeostasis, bile acid metabolism, FXR/RXR activation, and sphingomyelin metabolism and signaling. These findings are consistent with and build upon a growing body of evidence implicating certain PFAS in placental dysfunction via maternal vessel malperfusion, leading to increased risk for hypertensive disorders of pregnancy and related adverse pregnancy outcomes.

## Supplementary Material



## Data Availability

The data presented in the study can be accessed from the NTP data repository at https://doi.org/10.22427/NTP-DATA-109-003-003-000-6. Supplemental data can be found in the Supporting Information file.

## Notes

Methods, additional data related to the transcriptomic and qPCR analyses, pathway analyses, placental morphometry, and gene expression-phenotype correlations (PDF)
