# Supplementary material for: Transcriptomic Perturbations in Placental Gene Expression Following Developmental Exposure to Perfluorooctanoic Acid (PFOA) or Hexafluoropropylene Oxide-Dimer Acid (HFPO–DA or GenX) in CD‑1 Mice Are Consistent with Placental Insufficiency
Source: Environ Health (Wash). 2026 Mar 13;4(7):1417–32. doi: 10.1021/envhealth.5c00350 (PMC13386376; doi:10.1021/envhealth.5c00350)
Supplement: Supplementary file 1 [file eh5c00350_si_001.pdf]

Transcriptomic perturbations in placental gene expression following developmental exposure to perfluorooctanoic acid (PFOA) or hexafluoropropylene oxide-dimer acid (HFPO-DA or GenX) in CD-1 mice are consistent with placental insufficiency

Bevin E. Blake[1,2], Vesna A. Chappell[2], Colette N. Miller[1], Helen Nguyen[3], Trina P. Phan[2], Dhiral P. Phadke[4], Michele R. Balik-Meisner[4], Ignacio J. Tripodi[4], Deepak Mav[4], Ruchir R. Shah[4], Suzanne E. Fenton[5*]

[1]Public Health and Integrated Toxicology Division, Center for Public Health and Environmental Assessment, Office of Research and Development, U.S. Environmental Protection Agency, Research Triangle Park, NC, USA

[2]Mechanistic Toxicology Branch, Division of Translational Toxicology, National Institute of Environmental Health Sciences, Research Triangle Park, NC, USA

[3]Oak Ridge Institute for Science and Education, Center for Public Health and Environmental Assessment, U.S. Environmental Protection Agency, Research Triangle Park, NC, USA

[4]Sciome LLC, Research Triangle Park, NC, USA

[5]Center for Human Health and Environment, Department of Biological Sciences, North Carolina State University, Raleigh, N.C, USA

*Corresponding author email: sefenton@ncsu.edu

Appendix A: Detailed Methods

A.1 Animals

All samples were obtained during experiments previously performed in Blake et al. (2020)[11]. CD-1 mice were selected for this work for the following reasons: (1) CD-1 mice are more representative of the diversity of the human population as they are genetically outbred, (2) they produce large litters (*N* = 10-13) which requires fewer animals to achieve appropriate statistical power, (3) they are more health-protective in the context of human health risk assessment as they are more sensitive to PFAS than other mouse strains[19], and (4) they have been previously used in toxicological studies of PFOA and GenX in support of human health risk assessments[16, 20]. Two doses of PFOA were selected; a higher dose of 5 mg/kg/day which was previously shown to reduce birth weight and a lower dose of 1 mg/kg/day that does not induce the reduced birth weight phenotype in CD-1 mice[21]. We selected 10 mg/kg/day and 2 mg/kg/day GenX as "PFOA-equivalent" doses based on the two-fold difference between the U.S. EPA lifetime health advisory level for PFOA in drinking water of 70 ppt[20] and the provisional North Carolina state health goal for GenX of 140 ppt[22] (note that these health advisories and state health goals were selected during the design of the original experiment in 2018[11]). Oral administration of these doses resulted in comparable levels of PFOA or GenX in maternal serum at E 17.5 (1 mg/kg/day PFOA: 18.7 ± 3.2 µg PFOA/mL serum; 2 mg/kg/day GenX: 22.9 ± 17.1 µg GenX/mL serum; 5 mg/kg/day PFOA: 95.1 ± 14.1 µg PFOA/mL serum; 10 mg/kg/day GenX: 58.5 ± 34.6 µg GenX/mL serum)[11]. For PFOA, serum levels at the 1 mg/kg dose were within one order of magnitude of occupationally exposed populations in the US (median serum concentrations ranging from 0.58 to 5.71 ug/mL, reviewed by He et al. 2025)[23]. For GenX, serum levels at the 2 mg/kg dose were within three to four orders of magnitude for an occupationally exposed population (serum concentrations ranged from 0.0006 to 0.025 ug/mL)[24].

All tissues were obtained during experiments described in Blake et al. (2020)[11], which were approved by the NIEHS Animal Care and Use Committee (ASP #2017-0022). Briefly, mice were bred in-house during single overnight cohabitation and copulatory plug-positive mice were identified as embryonic day (E) 0.5. Pregnant CD-1 mice from the NIEHS colony were singly housed in humidity (45%-60%) and temperature (25°C)-controlled 12-hour light cycle rooms in polypropylene cages with *ad libitum* access to NIH-31 diet and reverse osmosis deionized (RODI)

water. Pregnant dams (2 blocks of equal size and total N = 10-13 per group) were exposed to 1 or 5 mg/kg perfluorooctanoic acid ammonium salt (PFOA, CAS #3825-26-1, Millipore Sigma, Burlington, MA, USA), 2 or 10 mg/kg GenX (ammonium 2,3,3,3-tetrafluoro-2-(heptafluoropropoxy) propanoate, CAS# 62037-80-3, SynQuest Laboratories, Alachua, FL, USA), or vehicle (RODI water) via daily oral gavage from E 1.5 to E 11.5 or E 17.5. The E 11.5 early gestation timepoint was selected as it overlaps a critical period of placental development in the mouse where the placenta undergoes vascularization with the uterine wall and chorioallantoic branching of vessels begins[25] while the later timepoint of E 17.5 was selected as the placenta is considered a mature organ at this stage. These timepoints allowed for the comparison of effects of chemical exposure in the immature/developing placenta (E 11.5) and mature/developed placenta (E 17.5).

A.2 Necropsy

Detailed necropsy procedures can be found in Blake et al. (2020)[11]. Briefly, on E 11.5 or 17.5 pregnant dams were weighed and humanely euthanized by swift decapitation and trunk blood was collected for internal dosimetry, as reported[11]. The uterus was removed and the placentas from viable embryos were collected in succession from the left uterine horn and immediately snap frozen in liquid nitrogen (*N* = 2-5 placentas/litter). For placentas collected at E 17.5, a portion of fetal tissue corresponding to each snap frozen placenta was reserved and genotyped by PCR amplification of the sex determining region-Y (SRY) gene (F: 5’ GCTTCAGTAATCTCAGCACCTAGAA 3’, R: 3’ CACATTGGCATGATAGCTCCAAATT 5’) to determine whether the fetus was genotypically male or female (TransnetYX, Inc). Sex determination was not conducted for placentas collected at E 11.5 as the embryo reproductive organs are not yet fully developed and thus not producing sex-specific steroids, thereby mitigating potential sex-specific effects[26]. By E 17.5, fetal gametes are fully developed and previous reports have shown important sex-specific differences in placental gene expression and response to exogenous insults [27].

A.3 RNA Isolation and Quantification

RNA isolation and quantification methods are reported in detail in our previous work[28]. In summary, a total of $N = 4–5$ placenta per treatment group, timepoint, and sex (E 17.5 only) were used in the current study. For placentas collected at E 11.5, three placentas per litter were pooled to achieve sufficient tissue mass for the RNA extraction protocol (> 0.5 mg). For placentas collected at E 17.5, no more than one placenta of each sex was used from a given litter. Snap frozen tissues were weighed then homogenized using a FastPrep-24™ 5G (MP Biomedicals, Valiant Co., Ltd, China) in Buffer RLT (Cat# 79216, Qiagen, Hilden, Germany) within 2mL, 2.4mm metal bead homogenization tubes (Thermo Fisher Scientific, Waltham, MA, USA). Homogenized placenta samples were snap frozen and stored at -80°C prior to RNA extraction. RNeasy kits were used following manufacturer's protocols to extract RNA from thawed samples (Cat# 75144, Qiagen, Hilden, Germany). Quality control of the resulting purified RNA was conducted using a NanoDrop™ 2000/2000c Spectrophotometer (ThermoFisher Scientific, Waltham, MA, USA) and Bioanalyzer High Sensitivity RNA Analysis (Agilent, Santa Clara, CA, USA). All RNA samples used for transcriptomics analysis had a 260/280 ratio > 2.0 and RIN > 5.0. RNA isolates were diluted to a standard concentration of 100 ng RNA/ 1 µL H_2O. RNA expression analysis was conducted using Affymetrix Mouse Clariom D arrays (Affymetrix, Santa Clara, CA, USA). Total RNA (25 ng/sample) was first amplified and labeled using the Affymetrix WT Plus Reagent Kit (WT Plus Kit) per manufacturer's instructions. Amplified biotin-cDNAs (5 µg/sample) were fragmented and hybridized to each array for 16 hours at 45°C in a rotating hybridization oven. Array slides were stained with streptavidin/phycoerythrin utilizing a double-antibody staining procedure and then washed for antibody amplification per the GeneChip Hybridization, Wash and Stain Kit user manual (Cat# 900720, ThermoFisher Scientific, Waltham, MA, USA). Arrays were scanned in an Affymetrix Scanner 3000 and data was obtained using Transcriptome Analysis Console Software (Affymetrix, Santa Clara, CA, USA).

A.4 Gene Expression Data Analyses

A detailed description of the transcriptomic analysis and workflow is described in Blake et al. (2022)[28] and raw data are accessible on NCBI GEO (accession number GSE262609). The probe

level raw intensity signal from the Affymetrix Mouse Clariom D Array (previously known as the Mouse Transcriptome Assay 1.0) was extracted. For each transcript these raw intensities were background adjusted, summarized using robust multi-array average (RMA) technique, and log2 transformed. The normalized expression was averaged across transcripts from the same gene to produce Entrez gene-level signal. Potential outliers were identified using a combination of approaches including principal component analysis, hierarchical cluster plots, and correlation plots resulting in one outlier detected and removed from the analyses. We utilized Student's t-test statistics to measure gene-level differential activity. A customized implementation of conventional Gene Set Enrichment Analysis (GSEA)[29] was employed to simultaneously identify differentially expressed genes (DEGs) and corresponding differentially enriched pathways (DEPs) for each chemical at each dose relative to the corresponding maternal or fetal vehicle control. Student's t-test statistics were used to measure gene-level differential activity and perform GSEA tests on all Hallmark pathways from the Molecular Signature Database (MSigDB, version 6.2) for which five or more genes were present in the microarray[29, 30]. A total of 50 pathways were used for the gene set enrichment analysis. The significant p-values, false detection rate, and familywise error rate (FWER) for gene/pathway level activity were computed using 10,000 random permutations of sample labels. False detection rate and FWER calculations were performed separately for all pathways. In the initial analysis, a gene was required to have an absolute fold change ≥ 2 and a distribution *P*-value ≤ 0.005 to be considered a significant DEG. However, these stringent parameters yielded very few genes, so a secondary analysis was performed using cutoffs of an absolute fold change ≥ 1.5 and a distribution *P*-value ≤ 0.05. The statistical cutoff used for a given figure or table is explicitly described in the legend or footnote. A significant DEP required an absolute Normalized Enrichment Score (NES) ≥ 1.5 and a NES *P*-value ≤ 0.05.

To further characterize the pathway-level differences between chemical exposure (PFOA or GenX) and placenta, gene-level results (fold change ≥ 1.5 and *P*-value ≤ 0.05) were imported into Ingenuity Pathway Analysis (IPA) software (Qiagen, Hilden, Germany) and analyzed using the Ingenuity Analysis Knowledgebase. A comparison analysis was performed to identify similarities and differences in canonical pathways and upstream regulators between the experimental groups and embryonic timepoints. The comparison analysis was visualized using heatmaps. IPA uses the right-tailed Fisher's Exact Test to calculate the *P*-value of overlap for identifying significant pathways. The *P*-values were then adjusted using the Benjamini-Hochberg

multiple testing correction method. Pathways with a -log(P-value) $\geq$ 1.3 were considered significantly activated.

We then used the DEG set identified using the less stringent criteria (absolute fold change ≥1.5 and $P \leq 0.05$) to explore the Gene Ontology (GO) Enrichment Analysis (PANTHER version 17.0, GO release 2023-01-01[31] The Gene Ontology Consortium, 2020). GO Enrichment Analysis was conducted first using the set of up-regulated DEGs identified across all treatment groups and time points in order to explore the biological processes associated with this suite of genes. GO Enrichment Analysis was conducted a second time using the set of down-regulated DEGs identified across all treatment groups and time points. Both analyses utilized PANTHER Overrepresentation Test using *Mus musculus* as the reference list and Fisher's Exact test with a False Discovery Rate (FDR) < 0.05.

A.5 Verification of Microarray Data

Placental RNA samples were used to verify the transcriptomics analysis. RNA (1µg) was transcribed into cDNA using High-Capacity cDNA synthesis kit (Applied Biosystems, Waltham, MA, USA). Complimentary DNA (12.5ng) was amplified for genes of interest in a QuantStudio 7 Flex Real Time PCR System (Applied Biosystems, Waltham, MA, USA) using PowerUP SYBR Green 2x mastermix (Applied Biosystems, Waltham, MA, USA). Primer sets for genes of interest were designed assays from IDT DNA (Coralville, IA, USA) and are presented in the supplemental materials section (**Table S1**). Relative mRNA levels were calculated by using ΔΔCt method and presented as fold change over respective vehicle control. Data is presented as fold change over vehicle control. One-way ANOVA, with Dunnett's multiple comparisons post hoc test (GraphPad Prism v.7), was performed to determine statistical significance between control and dose groups using ΔCt values (P-value ≤ 0.05) and 2-fold change or greater changes are presented as mean fold change for N=4-5, biological replicates, and visualized as a heatmap and P-value table.

A.6 Histopathology and placental morphometry

Additional detail on the histopathological methods and analyses performed on samples used in the present study are reported thoroughly in Foley et al[18]. Briefly, paraffin-embedded placentas were sectioned at 5 µm thickness and stained with hematoxylin and eosin (H&E)

according to standard protocols. Two trained histopathologists evaluated slides scanned at 40x using the Hamamatsu Nanozoomer S360 (Bridgewater, NJ, USA) and recorded consensus diagnoses, which included a severity score ranging from 0 = none, 1 = minimal, 2 = mild, 3 = moderate, 4 = marked, to 5 = severe.

For the morphometric analyses, only the E 17.5 timepoint was evaluated. Two trained experimenters blinded to the experimental conditions performed separate morphological assessments for the placental layers and decidual arteries ($N = 3–6$ placentas/group/sex). The total, decidual, and labyrinth area for each placenta was manually assessed using Aperio ImageScope software (Leica Biosystems). The junctional zone area was determined by calculating the difference between the measured areas. The decidual arterial wall thickness was calculated by determining the ratio of the total vessel area to the luminal area. Arteries were identified in the decidua region of the tissue through the presence of round endothelial cell nuclei, red blood cell abundance, thickened wall, and the overall circular shape of the vessel.

A.7 Gene expression-phenotype analysis

To determine whether specific genes or sets of genes were associated with phenotypic changes in the placenta, we performed analyses to evaluate relationships between gene expression and multiple placenta phenotypes. For each E 17.5 placenta with transcriptomic data, we looked at all littermates from the same dam and of the same sex. Continuous phenotypes (fetal weight, placenta weight, ratio of placenta to fetal weight, and placental morphometry) and histopathological severity scores were averaged across littermates.

Phenotypic endpoints were assessed to compare the 5 groups (vehicle control, GenX low dose (2 mg/kg/day GenX), GenX high dose (10 mg/kg/day GenX), PFOA low dose (1 mg/kg/day PFOA), and PFOA high dose (5 mg/kg/day PFOA). To directly compare each treatment group to the control group, the many-to-one (treatment groups to control group) Dunn Test was performed using a standard normal distribution. Endpoints with a treatment vs control P-value ≤ 0.05 were considered significant. To assess the relationship between differential gene expression and phenotype, a correlation analysis was performed. Pearson’s product moment correlation test was performed for each gene/phenotype combination to measure the degree of linear dependence between expression and phenotype. Unadjusted and adjusted (to control the false discovery rate)

P-values were calculated (Benjamini and Hochberg, 1995). Gene-phenotype pairs with correlations ≥ 0.7 and unadjusted *P*-values ≤ 0.05 were considered significant.

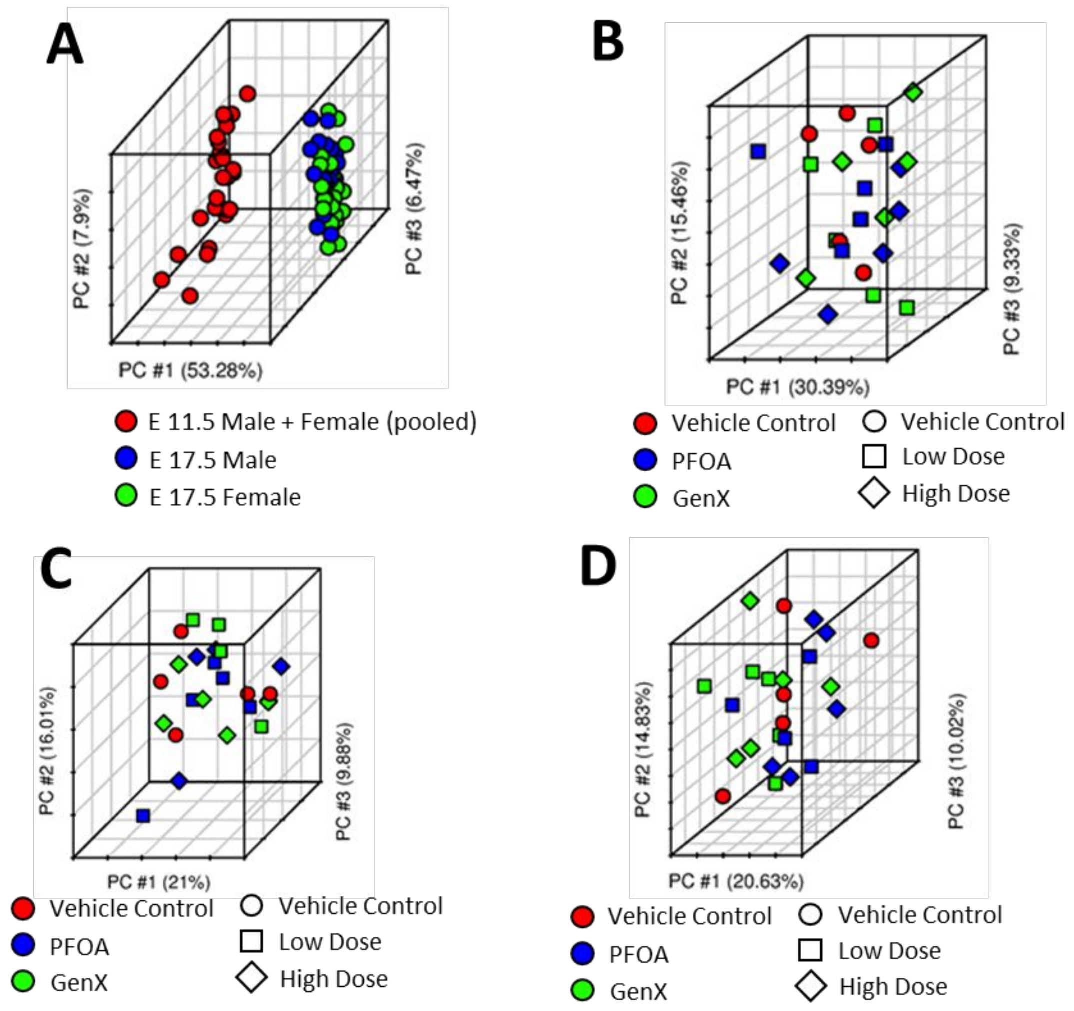

Figure S1. Principal components analysis of transcriptomic profiles for placenta showing separation of samples by timepoint (A) E 11.5 vs E 17.5, whereas samples did not exhibit a clear separation by treatment group or dose at E 11.5 (B) or E 17.5 for female placenta (C) or male placenta (D). Low Dose = 1 mg/kg/day for PFOA or 2 mg/kg/day for GenX, High Dose = 5 mg/kg/day PFOA or 10 mg/kg/day GenX.

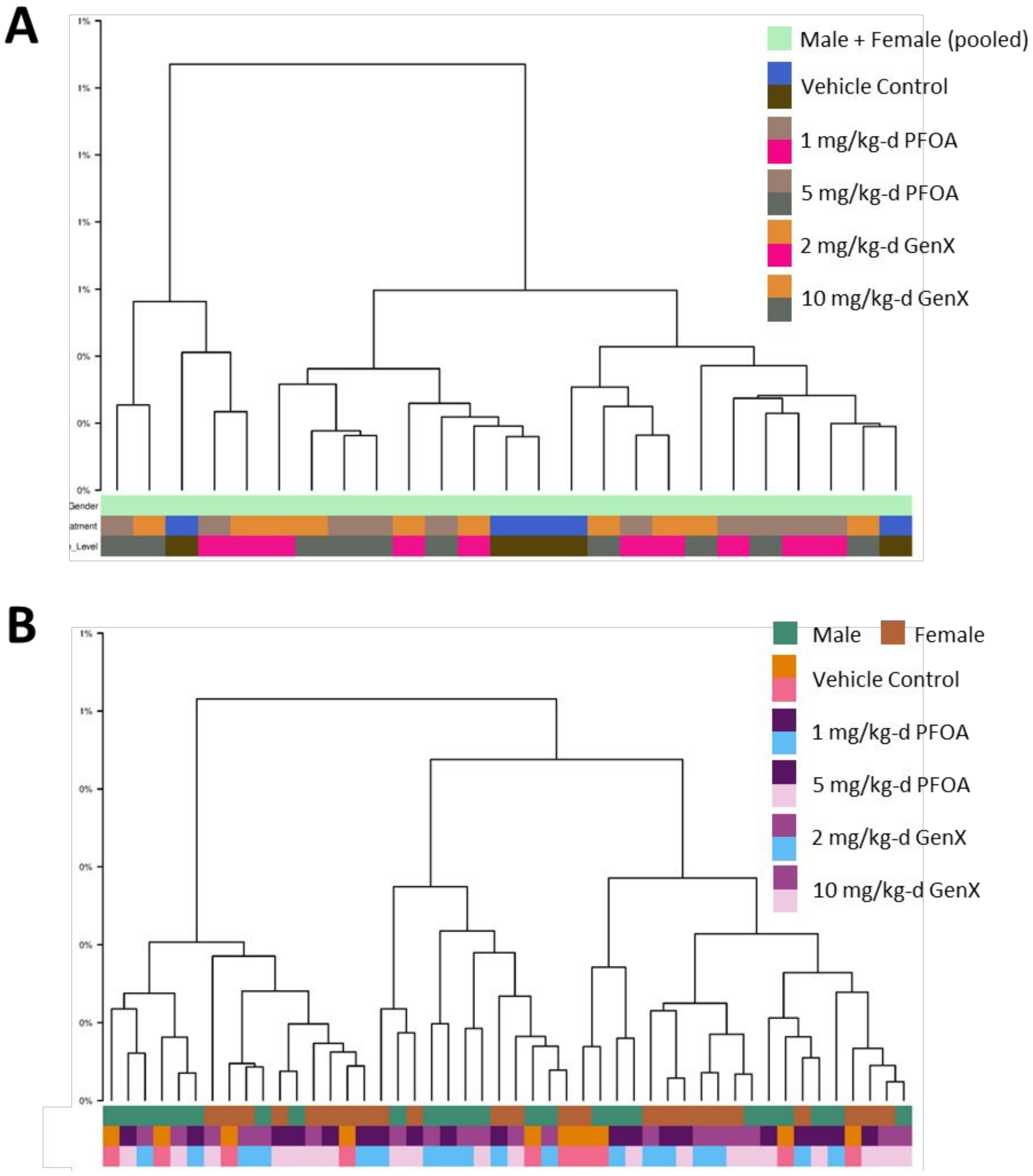

Figure S2. Hierarchical clustering of placenta transcriptomic profiles after outlier removal at E 11.5 (A) and E 17.5 (B). No obvious separation between treatment groups or doses were observed.

E11.5

	qPCR				Microarray			
	PFOA		GenX		PFOA		GenX	
	1 mg/kg	5 mg/kg	2 mg/kg	10 mg/kg	1 mg/kg	5 mg/kg	2 mg/kg	10 mg/kg
Cyp4a12b	3.09 *	-1.50	1.41	1.85	1.88	-1.10	1.03	1.26
Ear1	-1.09	3.32	2.31	1.14	1.66	7.05	3.86	1.26
Gkn2	-2.72 *	1.38	1.66	1.63	-1.60	1.21	-1.08	1.01
Hba-x	-1.17	-1.70 ‡	-1.53	-1.23	-1.38	-1.57	-1.49	-1.46
Prf1	1.76	1.49	2.53 ‡	2.32	1.59	1.54	1.85	1.66
Prl5a1	2.47 *	1.54	1.26	1.50	1.79	1.21	1.26	1.11
Scgb1a1	2.65	1.52	2.78 ‡	3.57 ‡	1.73	1.40	2.59	2.45
Tac2	6.00 *	2.13	3.40	5.87 *	1.75	1.18	1.41	2.08

-3 7

E17.5

	qPCR							
	Female				Male			
	PFOA		GenX		PFOA		GenX	
	1 mg/kg	5 mg/kg	2 mg/kg	10 mg/kg	1 mg/kg	5 mg/kg	2 mg/kg	10 mg/kg
Alb	1.22	-3.09	-29.30 *	-1.01	1.31	-2.64	3.83	2.22
Apoa2	-1.01	-2.36	-4.46 ‡	-1.70	-1.01	-2.60 *	1.51	1.59
C3	1.77	1.48	1.51	2.30 *	1.05	-1.15	2.69	-1.11
Ear1	-2.14 ‡	-3.10 *	-1.25	-2.51 *	-1.61	-2.93 *	-1.91 ‡	-1.82 *
GC	-1.02	-2.05	-6.73 *	-1.32	-2.02	-3.08	1.18	-1.19
Gypa	1.13	1.54	1.64 ‡	1.16	1.27	-1.02	1.32	1.15
Mmp12	-1.62	-3.64 *	-1.08	-2.01	1.09	-1.86	-1.02	-1.47
Rbp4	1.31	-1.97	-2.02	-1.34	-1.02	-2.21 *	1.18	1.02
Serpina1b	1.04	-2.28	-3.38	-1.59	-1.45	-2.63 *	1.13	-1.07
Smpd1	-1.66 ‡	-1.28	-1.90 *	-1.35	-1.46	-1.77	-1.73 ‡	-1.17

	Microarray							
	Female				Male			
	PFOA		GenX		PFOA		GenX	
	1 mg/kg	5 mg/kg	2 mg/kg	10 mg/kg	1 mg/kg	5 mg/kg	2 mg/kg	10 mg/kg
Alb	1.74	-1.05	-4.84	1.50	1.27	-1.70	1.60	1.01
Apoa2	1.11	-1.92	-2.85	-1.48	-1.43	-2.37	-1.29	-1.26
C3	1.24	1.38	1.33	1.74	1.76	1.01	1.41	1.39
Ear1	-2.59	-2.71	-1.28	-1.95	-2.21	-2.56	-2.18	-1.96
GC	1.08	-1.42	-2.73	-1.03	-1.34	-1.77	1.06	-1.13
Gypa	1.01	1.47	1.55	1.14	1.20	1.08	1.33	1.16
Mmp12	-1.34	-1.61	-1.05	-1.31	1.04	-1.22	-1.04	-1.21
Rbp4	1.33	-1.40	-1.34	-1.08	1.03	-1.65	1.03	-1.07
Serpina1b	1.35	-1.20	-1.57	1.01	-1.38	-1.73	1.03	-1.21
Smpd1	-1.46	-1.20	-1.56	-1.28	-1.47	-1.42	-1.57	-1.03

-29 3

Figure S3. Heatmap of select genes from E 11.5 and E 17.5 placenta transcriptomic microarray analysis and corresponding qPCR gene expression. Scale bars indicating the magnitude and direction of gene expression fold change are illustrated in the heatmap. Data is presented as mean fold change over control. Gray cells correspond to non-significant genes or genes with non-detectable levels of expression. Statistical cutoffs were applied to each gene: **P*-value<0.05, ‡*P*-value<0.1 and an absolute fold-change ≥1.5.

Table S1. IDT PrimeTime Primer Assay IDs

GOI	Target Name	TimePoint (E)	IDT Assay ID
Alb	Albumin	17.5	Mm.PT.58.6130903
Apoa2	Apolipoprotein A2	17.5	Mm.PT.58.43280476
C3	Complement C3	17.5	Mm.PT.58.17325540
Cyp4a12b	Cytochrome P450, family 4, subfamily a, polypeptide 12b	11.5	Mm.PT.58.41899199.g
Ear1	Eosinophil-associated ribonuclease A family 1	11.5	Mm.PT.58.31367994.g
GC	GC vitamin D binding protein	17.5	Mm.PT.58.29436747
Gkn2	Gastrokine 2	11.5	Mm.PT.56a.29739955
Gypa	Glycophorin A	17.5	Mm.PT.58.6297630
Hbz	Hemoglobin X, alpha-like embryonic chain in Hba	11.5	Mm.PT.58.10597504
Mmp12	Matrix metallopeptidase 12	17.5	Mm.PT.58.41952469
Prf1	Perforin 1	11.5	Mm.PT.58.41904164
Rbp4	Retinol binding protein 4	17.5	Mm.PT.58.30037794
Scgb1a1	Secretoglobin, family 1A, member 1	11.5	Mm.PT.58.6840684
Serpina1b	Serine peptidase inhibitor, clade A, member 1b	17.5	Mm.PT.58.8603196
Smpd1	Sphingomyelin phosphodiesterase 1	17.5	Mm.PT.58.10193202
Tac2	Tachykinin 2	11.5	Mm.PT.58.11219843
Prl3a1	Prolactin family 3, subfamily a, member 1	17.5	Mm.PT.56a.28423184
Prl8a6	Prolactin family 8, subfamily a, member 6	17.5	Mm.PT.58.17192859
Prl8a8	Prolactin family 8, subfamily a, member 8	17.5	Mm.PT.58.13974397
Prl5a1	Prolactin family 5, subfamily a, member 1	17.5	Mm.PT.58.32401996
Rpl19	Ribosomal protein L19	HKG	Mm.PT.58.12385796

Note: PrimeTime primer assays ordered from IDT.DNA.COM as primers only, for use with SYBR reagents. GOI = gene of interest. Mm = Mus musculus. HKG = housekeeping gene. Timepoint (E) = embryonic day.

Table S2. Number of significant* differentially expressed genes (DEGs) in placenta at embryonic day (E) 11.5 or 17.5 following exposure to PFOA or GenX.

		1 mg/kg PFOA	5 mg/kg PFOA	2 mg/kg GenX	10 mg/kg GenX
E 11.5	Up-regulated	29	35	7	5
	Down-regulated	2	1	1	0
	Total	31	36	8	5
E 17.5 Female	Up-regulated	0	3	12	1
	Down-regulated	4	6	4	6
	Total	4	9	16	7
E 17.5 Male	Up-regulated	0	1	3	2
	Down-regulated	6	15	6	5
	Total	6	16	9	7

*DEGs were considered significant if absolute fold-change values were ≥ 1.5 and $P \leq 0.05$

Table S3. Gene ontology biological process analysis for up-regulated DEGs identified in placentas exposed to PFOA or GenX

**GO biological process complete**	**Mus musculus - REFLIST (21997)**	**upload_1 (35)**	**upload_1 (expected)**	**upload_1 (over/under)**	**upload_1 (fold Enrichment)**	**upload_1 (raw P-value)**	**upload_1 (FDR)**
cell killing (GO:0001906)	144	5	0.23	+	21.82	3.65E-06	1.44E-02
regulation of response to biotic stimulus (GO:0002831)	370	6	0.59	+	10.19	2.54E-05	3.33E-02
regulation of defense response (GO:0031347)	655	8	1.04	+	7.68	7.39E-06	1.94E-02
innate immune response (GO:0045087)	897	9	1.43	+	6.31	8.69E-06	1.96E-02
response to bacterium (GO:0009617)	922	9	1.47	+	6.13	1.08E-05	1.89E-02
regulation of response to external stimulus (GO:0032101)	993	9	1.58	+	5.7	1.94E-05	2.79E-02
defense response to other organism (GO:0098542)	1171	10	1.86	+	5.37	1.01E-05	1.99E-02
response to other organism (GO:0051707)	1513	12	2.41	+	4.98	2.16E-06	3.41E-02
response to external biotic stimulus (GO:0043207)	1515	12	2.41	+	4.98	2.19E-06	1.73E-02
response to biotic stimulus (GO:0009607)	1554	12	2.47	+	4.85	2.85E-06	1.50E-02
biological process involved in interspecies interaction between organisms (GO:0044419)	1646	12	2.62	+	4.58	5.17E-06	1.63E-02
defense response (GO:0006952)	1554	11	2.47	+	4.45	1.90E-05	3.00E-02

Table S4. Gene ontology biological process analysis for down-regulated DEGs identified in placentas exposed to PFOA or GenX

GO biological process complete	Mus musculus - REFLIST (21997)	upload_1 (26)	upload_1 (expected)	upload_1 (over/under)	upload_1 (fold Enrichment)	upload_1 (raw P-value)	upload_1 (FDR)
response to methanol (GO:0033986)	4	2	0	+	> 100	2.00E-05	4.51E-02
response to chromate (GO:0046687)	5	2	0.01	+	> 100	2.80E-05	4.91E-02
negative regulation of serine-type peptidase activity (GO:1902572)	9	3	0.01	+	> 100	3.19E-07	5.03E-03
regulation of serine-type peptidase activity (GO:1902571)	9	3	0.01	+	> 100	3.19E-07	2.51E-03
negative regulation of serine-type endopeptidase activity (GO:1900004)	9	3	0.01	+	> 100	3.19E-07	1.68E-03
regulation of serine-type endopeptidase activity (GO:1900003)	9	3	0.01	+	> 100	3.19E-07	1.26E-03
hydrogen peroxide catabolic process (GO:0042744)	27	3	0.03	+	94	5.81E-06	1.83E-02
hydrogen peroxide metabolic process (GO:0042743)	43	3	0.05	+	59.03	2.14E-05	4.22E-02
negative regulation of biological process (GO:0048519)	5444	17	6.43	+	2.64	1.43E-05	3.76E-02

Table S5. Gene expression fold change and pvalues from qPCR gene expression analysis

	Pooled E 11.5				E 17.5 Female				E 17.5 Male			
GOI	PFOA 1 mg/kg	PFOA 5 mg/kg	GenX 2 mg/kg	GenX 10 mg/kg	PFOA 1 mg/kg	PFOA 5 mg/kg	GenX 2 mg/kg	GenX 10 mg/kg	PFOA 1 mg/kg	PFOA 5 mg/kg	GenX 2 mg/kg	GenX 10 mg/kg
Alb					1.22 (0.972)	**-3.09** (0.530)	**-29.30** (0.017)**	-1.01 (0.974)	1.31 (0.806)	**-2.64** (0.352)	**3.83** (0.714)	**2.22** (0.988)
Apoa2					-1.01 (0.999)	**-2.36** (0.245)	**-4.46** (0.058)*	**-1.70** (0.451)	-1.01 (0.992)	**-2.60** (0.022)**	**1.51** (0.779)	**1.59** (0.873)
C3					**1.77** (0.270)	1.48 (0.333)	**1.51** (0.253)	**2.30** **(0.045)**	1.05 (0.834)	1.15 (0.651)	**2.69** (0.285)	1.11 (0.588)
Cyp4a12b	**3.09** (0.027)**	-1.50 (0.406)	1.41 (0.922)	**1.85** (0.446)								
Ear1	-1.09 (0.999)	**3.32** (0.184)	**2.31** (0.847)	1.14 (0.995)	**-2.14** (0.073)*	**-3.10** (0.014)**	-1.25 (0.539)	**-2.51** (0.011)**	**-1.61** (0.112)	**-2.93** (0.012)**	**-1.91** *(0.062)*	**-1.82** (0.035)**
GC					-1.02 (0.948)	**-2.05** (0.324)	**-6.73** (0.031)**	-1.32 (0.734)	**-2.02** (0.582)	**-3.08** (0.141)	1.18 (0.868)	-1.19 (0.968)
Gkn2	**-2.72** (0.020)**	1.38 (0.404)	**1.66** (0.975)	**1.63** (0.639)								
Gypa					1.13 (0.910)	**1.54** (0.259)	**1.64** (0.089)*	1.16 (0.556)	1.27 (0.481)	-1.02 (0.981)	1.32 (0.955)	1.15 (0.865)
Hbz	-1.17 (0.551)	**-1.70** *(0.087)*	**-1.53** (0.121)	-1.23 (0.395)								
Mmp12					**-1.62** (0.369)	**-3.64** (0.017)**	-1.08 (0.997)	**-2.01** (0.237)	1.09 (0.992)	**-1.86** (0.124)	-1.02 (0.996)	-1.47 (0.223)
Prf1	**1.76** (0.647)	1.49 (0.678)	**2.53** (0.063)*	**2.32** (0.134)								
Prl5a1	**2.47** (0.038)**	**1.54** (0.290)	1.26 (0.751)	1.50 (0.957)								
Rbp4					1.31 (0.626)	**-1.97** (0.120)	**-2.02** (0.287)	-1.34 (0.526)	-1.02 (0.978)	**-2.21** (0.021)**	1.18 (0.857)	1.02 (0.922)
Scgb1a1	**2.65** (0.627)	**1.52** (0.710)	**2.78** (0.088)*	**3.57** (0.082)*								
Serpina1b					1.04 (0.950)	**-2.28** (0.418)	**-3.38** (0.199)	**-1.59** (0.809)	-1.45 (0.524)	**-2.63** (0.043)**	1.13 (1.000)	-1.07 (0.901)
Smpd1					**-1.66** (0.064)*	-1.28 (0.463)	**-1.90** (0.036)**	-1.35 (0.306)	-1.46 (0.312)	**-1.77** (0.113)	**-1.73** (0.059*)	-1.17 (0.880)
Tac2	**6.00** (0.040)**	**2.13** (0.565)	**3.40** (0.169)	**5.87** (0.017)**								

Note: Data presented as mean fold change over control (*P*-value). Statistical tests were performed using dCt values from ddCT method of calculating fold change. One-way ANOVA within a chemical group with a Dunnett's multiple comparison post hoc test.

***P*-values < 0.05, **P*-values < 0.1 are italicized. Absolute fold changes |> 1.5| are bolded. Grayed cells indicate genes that were not analyzed because they were not present in the microarray for that timepoint.

Table S6. Hallmark pathway normalized enrichment scores (*P*-value) in E 11.5 placentas after exposure to PFOA or GenX

Hallmark Pathway	1 mg/kg PFOA	5 mg/kg PFOA	2 mg/kg GenX	10 mg/kg GenX
Coagulation	1.29 (0.18)	0.57 (0.90)	0.69 (0.79)	1.22 (0.21)
Interferon alpha response	-0.85 (0.60)	0.83 (0.60)	1.17 (0.24)	1.31 (0.15)
Heme metabolism	-0.96 (0.47)	-0.71 (0.75)	0.82 (0.63)	-0.94 (0.47)
Cholesterol homeostasis	1.30 (0.17)	-0.4 (0.98)	0.77 (0.69)	0.73 (0.73)
Spermatogenesis	-1.41 (0.10)	-1.48 (0.06)	-1.54 (0.04)*	-1.51 (0.05)
Hedgehog signaling	1.56 (0.04)*	-0.73 (0.72)	1.00 (0.41)	1.10 (0.31)
Bile acid metabolism	1.11 (0.31)	-0.85 (0.57)	-0.79 (0.66)	-1.06 (0.34)
Pancreas beta cells	0.54 (0.93)	-1.57 (0.04)*	-1.38 (0.10)	-0.79 (0.66)
Oxidative phosphorylation	0.69 (0.79)	1.45 (0.07)	1.27 (0.16)	-0.70 (0.76)
KRAS signaling down	-0.81 (0.65)	-1.62 (0.02)	-1.29 (0.15)	-0.62 (0.86)
Protein secretion	0.73 (0.74)	1.38 (0.11)	1.55 (0.03)*	0.77 (0.68)
Adipogenesis	1.05 (0.37)	1.24 (0.19)	1.41 (0.09)	0.67 (0.80)
TGF beta signaling	0.74 (0.73)	1.18 (0.24)	1.41 (0.09)	1.14 (0.28)
Estrogen response late	0.87 (0.58)	-0.86 (0.57)	0.79 (0.67)	1.04 (0.36)
Hypoxia	1.14 (0.29)	0.93 (0.49)	1.42 (0.08)	1.69 (0.01)*
Epithelial mesenchymal transition	1.17 (0.26)	-0.49 (0.95)	0.86 (0.58)	1.40 (0.10)
Peroxisome	1.13 (0.30)	0.77 (0.69)	1.03 (0.37)	0.81 (0.62)
Allograft rejection	0.67 (0.82)	-0.87 (0.55)	-0.87 (0.56)	1.36 (0.12)
IL6 JAK STAT3 signaling	-0.82 (0.63)	0.77 (0.68)	1.29 (0.15)	1.53 (0.04)
Fatty acid metabolism	1.35 (0.14)	0.84 (0.60)	1.19 (0.22)	0.71 (0.75)
Angiogenesis	1.34 (0.14)	0.88 (0.54)	1.31 (0.14)	1.38 (0.11)
MYC targets V2	-0.55 (0.92)	0.88 (0.55)	-0.33 (1.00)	-1.13 (0.28)
MYC targets V1	-0.34 (0.99)	1.08 (0.34)	0.25 (1.00)	-1.14 (0.27)
Unfolded protein response	0.47 (0.97)	1.46 (0.07)	1.02 (0.39)	-0.56 (0.90)
Inflammatory response	-0.72 (0.75)	-0.66 (0.81)	0.94 (0.48)	1.46 (0.07)
Complement	0.91 (0.54)	0.89 (0.53)	1.07 (0.33)	1.16 (0.26)
TNFA signaling via NFKB	-0.56 (0.91)	0.75 (0.71)	1.15 (0.26)	1.50 (0.05)
PI3K AKT MTOR signaling	-0.77 (0.69)	1.17 (0.26)	1.06 (0.34)	-0.71 (0.75)
WNT beta catenin signaling	0.85 (0.60)	-1.01 (0.40)	0.58 (0.91)	-0.66 (0.81)
Myogenesis	-0.67 (0.81)	-1.07 (0.34)	-0.93 (0.48)	1.03 (0.37)
UV response up	-0.81 (0.64)	-0.72 (0.74)	0.86 (0.58)	0.83 (0.60)
E2F targets	-0.37 (0.99)	0.29 (1.00)	-0.55 (0.93)	-1.43 (0.08)
MTORC1 signaling	0.97 (0.47)	1.09 (0.33)	0.85 (0.59)	-0.58 (0.89)
Apical surface	0.72 (0.76)	-1.19 (0.23)	0.88 (0.55)	1.16 (0.25)
Apoptosis	0.94 (0.50)	0.92 (0.50)	1.22 (0.19)	1.49 (0.06)
Apical jucntion	0.89 (0.55)	-0.99 (0.42)	0.86 (0.58)	0.99 (0.40)
Glycolysis	1.34 (0.14)	1.02 (0.40)	1.35 (0.11)	0.99 (0.41)
Androgen response	0.84 (0.61)	0.83 (0.61)	0.70 (0.78)	0.69 (0.77)
Estrogen response early	-0.91 (0.53)	-1.00 (0.41)	0.72 (0.75)	0.97 (0.43)
IL2 STAT5 signaling	0.91 (0.53)	0.69 (0.77)	1.11 (0.30)	1.35 (0.13)
DNA repair	-0.75 (0.72)	1.31 (0.15)	0.97 (0.45)	-0.87 (0.55)
P53 pathway	-0.81 (0.65)	1.01 (0.40)	1.20 (0.22)	1.27 (0.18)
G2M checkpoint	0.24 (1.00)	0.31 (0.99)	-0.43 (0.98)	-1.32 (0.14)
NOTCH signaling	0.86 (0.60)	0.45 (0.97)	1.16 (0.25)	0.92 (0.48)
KRAS signaling up	1.11 (0.31)	0.67 (0.80)	1.13 (0.28)	1.22 (0.21)
Reactive oxygen species pathway	0.78 (0.68)	0.88 (0.54)	0.80 (0.65)	0.73 (0.73)
Interferon gamma response	-0.56 (0.92)	0.63 (0.83)	1.04 (0.36)	1.44 (0.08)
Mitotic spindle	0.42 (0.98)	1.11 (0.30)	0.92 (0.50)	-0.94 (0.47)
Xenobiotic metabolism	1.36 (0.13)	0.84 (0.59)	0.90 (0.52)	1.03 (0.36)
UV response down	1.11 (0.31)	1.04 (0.37)	1.31 (0.14)	1.10 (0.30)

Table S7. Hallmark pathway normalized enrichment scores (*P*-value) in E 17.5 female placentas after exposure to PFOA or GenX

Hallmark Pathway	1 mg/kg PFOA	5 mg/kg PFOA	2 mg/kg GenX	10 mg/kg GenX
Coagulation	-0.68 (0.86)	-0.85 (0.65)	-1.71 (0.004)*	-0.61 (0.92)
Interferon alpha response	-0.63 (0.91)	1.4 (0.07)	0.82 (0.72)	0.77 (0.74)
Heme metabolism	0.74 (0.79)	1.69 (0.01)*	1.29 (0.15)	1.65 (0.01)*
Cholesterol homeostasis	0.69 (0.84)	0.88 (0.61)	-1.84 (0.007)*	1.47 (0.05)
Spermatogenesis	-0.77 (0.74)	-0.93 (0.54)	0.66 (0.91)	1.24 (0.17)
Hedgehog signaling	-1.08 (0.33)	-1.13 (0.29)	-0.93 (0.55)	-0.86 (0.60)
Bile acid metabolism	1.05 (0.37)	0.85 (0.66)	-1.5 (0.04)*	1.25 (0.16)
Pancreas beta cells	-0.76 (0.75)	-1.12 (0.30)	-0.86 (0.65)	0.46 (0.99)
Oxidative phosphorylation	0.89 (0.58)	1.57 (0.02)*	0.87 (0.64)	1.16 (0.23)
KRAS signaling down	-0.47 (0.99)	-1.15 (0.26)	-0.85 (0.65)	-0.52 (0.98)
Protein secretion	-0.65 (0.89)	1.08 (0.34)	0.51 (0.98)	0.43 (0.99)
Adipogenesis	0.92 (0.53)	1.56 (0.02)*	0.7 (0.87)	1.21 (0.19)
TGF beta signaling	0.91 (0.55)	1.53 (0.03)*	1.19 (0.23)	1.03 (0.37)
Estrogen response late	-0.99 (0.44)	-0.77 (0.79)	-1.13 (0.28)	0.76 (0.75)
Hypoxia	-1.05 (0.36)	0.93 (0.54)	0.97 (0.49)	0.71 (0.82)
Epithelial mesenchymal transition	-1.11 (0.30)	-1.27 (0.15)	-0.84 (0.67)	-1.41 (0.07)
Peroxisome	1.07 (0.34)	1.39 (0.08)	-1.24 (0.18)	1.62 (0.02)*
Allograft rejection	-1.51 (0.04)	-0.99 (0.45)	-0.75 (0.81)	0.78 (0.72)
IL6 JAK STAT3 signaling	-1.13 (0.27)	0.88 (0.60)	0.70 (0.86)	-0.63 (0.91)
Fatty acid metabolism	1.13 (0.28)	1.48 (0.04)*	-1.09 (0.33)	1.54 (0.03)*
Angiogenesis	-0.65 (0.89)	-0.82 (0.70)	0.81 (0.73)	-0.85 (0.62)
MYC targets V2	0.48 (0.98)	1.53 (0.03)*	-0.51 (0.99)	1.02 (0.39)
MYC targets V1	0.64 (0.91)	1.51 (0.04)	1.16 (0.26)	0.97 (0.45)
Unfolded protein response	-1.09 (0.32)	0.99 (0.46)	-0.42 (1.00)	-0.48 (0.99)
Inflammatory response	-1.09 (0.32)	-0.72 (0.84)	-0.95 (0.52)	-0.70 (0.83)
Complement	-0.80 (0.70)	0.70 (0.87)	-1.10 (0.32)	0.97 (0.45)
TNFA signaling via NFKB	-0.82 (0.67)	0.8 (0.74)	0.81 (0.73)	-0.81 (0.68)
PI3K AKT MTOR signaling	-0.69 (0.84)	0.99 (0.45)	0.89 (0.61)	0.95 (0.47)
WNT beta catenin signaling	1.15 (0.25)	0.66 (0.90)	1.11 (0.31)	0.87 (0.59)
Myogenesis	0.87 (0.59)	-0.99 (0.46)	-0.82 (0.71)	-0.70 (0.83)
UV response up	-0.90 (0.56)	1.10 (0.32)	-0.93 (0.54)	1.11 (0.28)
E2F targets	-0.66 (0.88)	0.94 (0.53)	0.58 (0.96)	0.88 (0.58)
MTORC1 signaling	-0.90 (0.56)	1.30 (0.13)	-0.91 (0.57)	0.83 (0.65)
Apical surface	0.59 (0.94)	-0.98 (0.47)	-1.04 (0.40)	-0.93 (0.50)
Apoptosis	0.75 (0.77)	1.04 (0.39)	0.84 (0.68)	0.83 (0.65)
Apical jucntion	0.75 (0.76)	-0.84 (0.67)	1.23 (0.19)	0.76 (0.75)
Glycolysis	-1.04 (0.38)	0.97 (0.49)	-0.93 (0.55)	0.96 (0.46)
Androgen response	-0.68 (0.85)	1.22 (0.19)	-0.86 (0.64)	0.93 (0.50)
Estrogen response early	0.93 (0.52)	0.86 (0.63)	-1.05 (0.38)	0.99 (0.42)
IL2 STAT5 signaling	0.85 (0.62)	0.97 (0.48)	1.22 (0.20)	0.91 (0.52)
DNA repair	-0.59 (0.94)	1.33 (0.11)	0.71 (0.86)	0.82 (0.67)
P53 pathway	1.02 (0.40)	1.18 (0.24)	1.08 (0.35)	1.13 (0.27)
G2M checkpoint	-0.52 (0.97)	1.02 (0.41)	0.65 (0.92)	0.79 (0.71)
NOTCH signaling	1.18 (0.23)	1.03 (0.41)	1.48 (0.04)*	-0.88 (0.58)
KRAS signaling up	-0.84 (0.63)	-0.94 (0.52)	-0.85 (0.65)	-0.86 (0.61)
Reactive oxygen species pathway	-0.58 (0.95)	1.16 (0.25)	-0.78 (0.77)	0.82 (0.66)
Interferon gamma response	-0.93 (0.52)	1.17 (0.24)	0.67 (0.90)	0.78 (0.73)
Mitotic spindle	0.84 (0.63)	1.20 (0.21)	1.18 (0.24)	0.97 (0.45)
Xenobiotic metabolism	-0.94 (0.50)	0.91 (0.57)	-1.46 (0.05)	1.05 (0.36)
UV response down	1.16 (0.24)	1.23 (0.18)	1.36 (0.10)	0.73 (0.79)

Table S8. Hallmark pathway normalized enrichment scores (*P*-value) in E 17.5 male placentas after exposure to PFOA or GenX

Hallmark Pathway	1 mg/kg PFOA	5 mg/kg PFOA	2 mg/kg GenX	10 mg/kg GenX
Coagulation	-0.88 (0.58)	-1.90 (0.005)*	0.73 (0.76)	-0.77 (0.75)
Interferon alpha response	0.65 (0.90)	1.04 (0.37)	1.65 (0.01)*	1.67 (0.01)*
Heme metabolism	-0.71 (0.82)	0.74 (0.78)	1.04 (0.35)	1.03 (0.38)
Cholesterol homeostasis	-1.44 (0.06)	-1.56 (0.02)*	0.79 (0.68)	1.03 (0.38)
Spermatogenesis	0.72 (0.82)	0.91 (0.53)	-0.92 (0.50)	-1.24 (0.16)
Hedgehog signaling	-0.74 (0.79)	-1.25 (0.17)	-1.13 (0.26)	-1.21 (0.19)
Bile acid metabolism	-1.03 (0.38)	-1.37 (0.09)	-0.72 (0.78)	-0.73 (0.81)
Pancreas beta cells	0.84 (0.63)	-0.82 (0.67)	-0.56 (0.95)	-0.76 (0.77)
Oxidative phosphorylation	-1.07 (0.34)	0.86 (0.61)	1.33 (0.11)	1.46 (0.04)*
KRAS signaling down	0.62 (0.93)	-0.57 (0.95)	-1.28 (0.14)	-1.21 (0.20)
Protein secretion	-1.41 (0.07)	-0.78 (0.73)	0.98 (0.43)	1.38 (0.08)
Adipogenesis	-1.20 (0.22)	-0.68 (0.86)	1.05 (0.35)	0.94 (0.49)
TGF beta signaling	-1.01 (0.40)	-1.03 (0.37)	-0.51 (0.98)	-0.65 (0.90)
Estrogen response late	-1.45 (0.06)	-1.63 (0.01)*	-1.05 (0.34)	-1.15 (0.24)
Hypoxia	-1.19 (0.23)	-1.11 (0.30)	0.63 (0.89)	-1.28 (0.14)
Epithelial mesenchymal transition	-0.88 (0.58)	-1.88 (0.009)*	-1.04 (0.35)	-1.45 (0.05)
Peroxisome	-0.84 (0.64)	-0.98 (0.44)	1.06 (0.34)	1.06 (0.34)
Allograft rejection	-0.88 (0.58)	-1.16 (0.25)	-0.92 (0.50)	-0.91 (0.54)
IL6 JAK STAT3 signaling	-1.18 (0.24)	-1.31 (0.13)	-0.73 (0.77)	0.65 (0.90)
Fatty acid metabolism	-1.19 (0.23)	-0.82 (0.67)	0.97 (0.44)	0.87 (0.60)
Angiogenesis	0.82 (0.67)	-1.57 (0.02)*	0.50 (0.98)	-0.92 (0.53)
MYC targets V2	-0.75 (0.77)	1.29 (0.14)	-0.57 (0.95)	1.08 (0.31)
MYC targets V1	-0.46 (0.99)	1.26 (0.17)	1.16 (0.24)	1.46 (0.04)*
Unfolded protein response	-1.32 (0.12)	-0.58 (0.95)	-0.60 (0.92)	0.60 (0.94)
Inflammatory response	-0.89 (0.56)	-1.26 (0.16)	-0.91 (0.52)	-0.89 (0.57)
Complement	-1.13 (0.28)	-1.29 (0.14)	0.91 (0.53)	0.80 (0.71)
TNFA signaling via NFKB	-1.20 (0.21)	-1.14 (0.27)	-0.95 (0.47)	-1.08 (0.32)
PI3K AKT MTOR signaling	-1.30 (0.13)	0.77 (0.74)	0.64 (0.88)	0.91 (0.54)
WNT beta catenin signaling	-0.98 (0.44)	-0.79 (0.71)	-1.02 (0.38)	-0.83 (0.66)
Myogenesis	-0.92 (0.52)	-1.42 (0.07)	-1.11 (0.28)	-1.26 (0.15)
UV response up	-1.11 (0.29)	0.79 (0.71)	0.78 (0.69)	-0.60 (0.94)
E2F targets	-0.49 (0.98)	1.10 (0.32)	0.69 (0.82)	1.02 (0.39)
MTORC1 signaling	-1.34 (0.11)	-0.78 (0.72)	0.93 (0.49)	1.12 (0.28)
Apical surface	-0.86 (0.62)	-1.22 (0.19)	-1.06 (0.34)	-0.98 (0.44)
Apoptosis	-1.05 (0.36)	-1.25 (0.17)	0.69 (0.81)	-0.68 (0.87)
Apical jucntion	-0.81 (0.69)	-1.31 (0.13)	0.55 (0.96)	-1.01 (0.40)
Glycolysis	-1.15 (0.26)	-1.00 (0.41)	0.83 (0.62)	0.73 (0.81)
Androgen response	-1.23 (0.19)	-0.82 (0.67)	0.88 (0.56)	1.08 (0.32)
Estrogen response early	-1.04 (0.37)	-1.10 (0.31)	-0.94 (0.47)	-0.94 (0.50)
IL2 STAT5 signaling	-1.04 (0.37)	-1.19 (0.22)	0.69 (0.82)	-0.75 (0.77)
DNA repair	-1.01 (0.41)	0.86 (0.61)	0.73 (0.77)	1.03 (0.37)
P53 pathway	-0.90 (0.55)	-0.90 (0.56)	-0.64 (0.88)	-0.54 (0.97)
G2M checkpoint	-0.52 (0.97)	0.80 (0.70)	0.70 (0.81)	1.16 (0.23)
NOTCH signaling	-0.60 (0.93)	-0.94 (0.49)	-0.62 (0.90)	-0.93 (0.51)
KRAS signaling up	-0.68 (0.86)	-1.49 (0.04)*	-0.72 (0.78)	-0.86 (0.60)
Reactive oxygen species pathway	-1.16 (0.25)	-1.10 (0.31)	1.10 (0.29)	1.16 (0.23)
Interferon gamma response	0.64 (0.91)	0.77 (0.74)	1.38 (0.09)	1.34 (0.10)
Mitotic spindle	-0.67 (0.87)	0.71 (0.82)	0.90 (0.53)	1.05 (0.35)
Xenobiotic metabolism	-1.20 (0.22)	-1.28 (0.14)	1.04 (0.36)	0.95 (0.48)
UV response down	-0.75 (0.77)	-0.93 (0.50)	-0.72 (0.79)	-0.60 (0.94)

Table S9. E 17.5 female placenta morphometry (mean ± SD)

	Control	1 mg/kg PFOA	5 mg/kg PFOA	2 mg/kg GenX	10 mg/kg GenX
N	5	5	5	6	4
Total Placenta Area (um^2)	1.25E+07 ± 1.55E+06	1.27E+07 ± 8.18E+05	1.40E+07 ± 1.33E+06	1.24E+07 ± 1.59E+06	1.38E+07 ± 1.46E+06
Decidua (um^2)	2.07E+06 ± 2.69E+05	1.83E+06 ± 3.86E+05	1.88E+06 ± 3.26E+05	1.88E+06 ± 5.94E+05	1.55E+06 ± 2.51E+05
Labyrinth (um^2)	7.83E+06 ± 1.14E+06	8.33E+06 ± 4.21E+05	**8.93E+06 ± 4.87E+05***	8.19E+06 ± 6.14E+05	**9.75E+06 ± 7.24E+05***
Junctional Zone (um^2)	2.61E+06 ± 4.57E+05	2.51E+06 ± 3.66E+05	3.16E+06 ± 7.17E+05	2.33E+06 ± 9.27E+05	2.45E+06 ± 1.15E+06
Decidua (% total area)	16.64 ± 2.42	14.36 ± 2.36	**13.42 ± 1.15***	14.91 ± 3.35	**11.27 ± 1.29***
Labyrinth (% total area)	62.52 ± 3.10	65.83 ± 3.19	64.17 ± 3.99	66.72 ± 7.86	**71.36 ± 7.60***
Junctional Zone (% total area)	20.84 ± 2.33	19.81 ± 2.51	22.42 ± 3.20	18.36 ± 5.56	17.37 ± 7.04
Labyrinth/Junctional Zone Ratio	3.04 ± 0.48	3.38 ± 0.56	2.93 ± 0.57	4.02 ± 1.60	5.02 ± 3.09
Decidua/Junctional Zone Ratio	0.81 ± 0.16	0.74 ± 0.15	0.60 ± 0.07	0.84 ± 0.17	0.76 ± 0.41
Labyrinth/Decidua Ratio	3.83 ± 0.62	4.70 ± 0.91	4.83 ± 0.69	4.77 ± 1.63	**6.43 ± 1.28***
Average vessel area (um^2)	2.73E+04 ± 7.75E+03	2.42E+04 ± 7.26E+03	3.54E+04 ± 2.80E+04	3.43E+04 ± 1.57E+04	2.72E+04 ± 1.33E+04
Average luminal area (um^2)	8.82E+03 ± 3.55E+03	1.05E+04 ± 6.90E+03	1.49E+04 ± 1.20E+04	1.30E+04 ± 6.70E+03	1.02E+04 ± 5.13E+03
Average luminal length (um)	448.43 ± 111.51	469.30 ± 122.42	485.66 ± 135.69	500.13 ± 167.74	476.38 ± 150.17
Average vessel:luminal area	2.72 ± 0.59	3.27 ± 0.54	2.79 ± 0.64	2.47 ± 0.36	2.70 ± 0.93

*P<0.05 by ANOVA

Table S10. E 17.5 male placenta morphometry (mean ± SD)

	Control	1 mg/kg PFOA	5 mg/kg PFOA	2 mg/kg GenX	10 mg/kg GenX
N	5	4	6	3	5
Total Placenta Area (um^2)	1.17E+07 ± 2.11E+06	1.28E+07 ± 1.67E+06	**1.47E+07 ± 1.75E+06***	1.40E+07 ± 3.70E+06	1.41E+07 ± 6.88E+05
Decidua (um^2)	2.06E+06 ± 4.50E+05	1.91E+06 ± 4.05E+05	1.89E+06 ± 5.18E+05	2.38E+06 ± 7.99E+05	1.97E+06 ± 1.43E+05
Labyrinth (um^2)	7.01E+06 ± 1.25E+06	8.37E+06 ± 1.18E+06	**9.44E+06 ± 9.09E+05***	8.48E+06 ± 8.36E+05	**8.88E+06 ± 7.85E+05***
Junctional Zone (um^2)	2.61E+06 ± 9.05E+05	2.54E+06 ± 4.34E+05	3.36E+06 ± 1.00E+06	3.18E+06 ± 2.29E+06	3.22E+06 ± 1.42E+06
Decidua (% total area)	17.61 ± 1.60	14.81 ± 2.01	**12.81 ± 3.17***	16.81 ± 3.11	**14.00 ± 1.00***
Labyrinth (% total area)	60.21 ± 4.46	65.29 ± 3.36	64.52 ± 4.30	62.30 ± 11.89	63.45 ± 8.01
Junctional Zone (% total area)	22.18 ± 5.65	19.90 ± 3.14	22.67 ± 4.76	20.89 ± 10.12	22.56 ± 8.55
Labyrinth/Junctional Zone Ratio	2.86 ± 0.70	3.36 ± 0.67	2.97 ± 0.69	3.79 ± 2.65	3.14 ± 1.11
Decidua/Junctional Zone Ratio	0.84 ± 0.22	0.76 ± 0.15	0.60 ± 0.27	0.92 ± 0.35	0.68 ± 0.21
Labyrinth/Decidua Ratio	3.43 ± 0.24	4.47 ± 0.65	**5.27 ± 1.23***	3.86 ± 1.42	4.54 ± 0.54
Average vessel area (um^2)	4.26E+04 ± 2.91E+04	3.89E+04 ± 3.32E+04	3.35E+04 ± 1.27E+04	2.87E+04 ± 1.48E+04	2.65E+04 ± 1.62E+04
Average luminal area (um^2)	1.40E+04 ± 8.99E+03	1.40E+04 ± 9.40E+03	1.21E+04 ± 7.26E+03	8.12E+03 ± 4.54E+03	1.07E+04 ± 6.64E+03
Average luminal length (um)	446.85 ± 129.16	515.88 ± 148.63	398.09 ± 112.14	461.25 ± 121.01	511.58 ± 182.47
Average vessel:luminal area	2.45 ± 0.53	3.00 ± 0.37	**3.69 ± 0.30***	3.10 ± 1.16	2.44 ± 0.71

*P<0.05 by ANOVA

Table S11. Number of genes significantly correlated with placental and fetal endpoints.

Endpoint category	Placenta region(s)	Endpoint name	E 17.5 Female	E 17.5 Male
Fetal-Placental measures	Whole placenta	Fetal weight	6	5
Fetal-Placental measures	Whole placenta	Placental weight	8	25
Fetal-Placental measures	Whole placenta	Placental:Fetal weight ratio	45	27
Placental histopathology	Junctional zone	Angiectasis, junctional zone	31	31
Placental histopathology	Junctional zone	Coagulative spongiotrophoblast necrosis	109	56
Placental histopathology	Junctional zone	Glycogen cell lacunae, acellular basophilic contents	19	32
Placental histopathology	Junctional zone	Glycogen cell lacunae, minimal acellular contents	24	14
Placental histopathology	Junctional zone	Mineralization	7	8
Placental histopathology	Labyrinth	Atrophy	22	27
Placental histopathology	Labyrinth	Congestion	24	24
Placental histopathology	Labyrinth	Cystic degeneration	8	26
Placental histopathology	Labyrinth	Maternal sinus dilation	15	24
Placental histopathology	Labyrinth	Necrosis	6	3
Placental histopathology	Decidua	Necrosis	10	8
Placental histopathology	Decidua	Spiral artery fibrinoid necrosis	6	18
Placental histopathology	Decidua	Thrombosis	8	7
Placental morphometry	Decidua	Average vessel:luminal area ratio	37	27
Placental morphometry	Decidua	Relative decidua size	17	61
Placental morphometry	Decidua and Junctional Zone	Decidua:Junctional zone relative size ratio	10	104
Placental morphometry	Junctional zone	Junctional zone size (relative)	4	23
Placental morphometry	Labyrinth	Labyrinth size (relative)	9	7
Placental morphometry	Labyrinth and Decidua	Labyrinth:Decidua relative size ratio	18	23
Placental morphometry	Labyrinth and Junctional Zone	Labyrinth:Junctional zone relative size ratio	8	23
		**Total**	**451**	**603**

Note: Numerical histopathology severity scores were used to evaluate the correlation between gene expression and phenotype and included the following potential scores: 0 = none, 1 = minimal, 2 = mild, 3 = moderate, 4 = marked, 5 = severe.
